# “Into and Out of” the Qinghai‐Tibet Plateau and the Himalayas: Centers of origin and diversification across five clades of Eurasian montane and alpine passerine birds

**DOI:** 10.1002/ece3.6615

**Published:** 2020-08-04

**Authors:** Martin Päckert, Adrien Favre, Jan Schnitzler, Jochen Martens, Yue‐Hua Sun, Dieter Thomas Tietze, Frank Hailer, Ingo Michalak, Patrick Strutzenberger

**Affiliations:** ^1^ Senckenberg Natural History Collections, Museum of Zoology Dresden Germany; ^2^ Entomology III Senckenberg Research Institute and Natural History Museum Frankfurt Frankfurt am Main Germany; ^3^ German Centre for Integrative Biodiversity Research (iDiv) Halle‐Jena‐Leipzig Leipzig Germany; ^4^ Department of Molecular Evolution and Plant Systematics & Herbarium (LZ) Institute of Biology Leipzig University Leipzig Germany; ^5^ Institute of Organismic and Molecular Evolution Johannes Gutenberg‐Universität Mainz Germany; ^6^ Key Laboratory of Animal Ecology and Conservation Institute of Zoology Chinese Academy of Sciences Beijing China; ^7^ Natural History Museum Basel Basel Switzerland; ^8^ Centrum für Naturkunde Universität Hamburg Hamburg Germany; ^9^ School of Biosciences Cardiff University Cardiff UK; ^10^ Senckenberg Biodiversity and Climate Research Centre Frankfurt am Main Germany; ^11^ Department of Botany and Biodiversity Research Universität Wien Wien Austria

**Keywords:** ancestral ranges, center of origin, immigration, in situ diversification, Qinghai‐Tibet Plateau, Sinohimalayas

## Abstract

Encompassing some of the major hotspots of biodiversity on Earth, large mountain systems have long held the attention of evolutionary biologists. The region of the Qinghai‐Tibet Plateau (QTP) is considered a biogeographic source for multiple colonization events into adjacent areas including the northern Palearctic. The faunal exchange between the QTP and adjacent regions could thus represent a one‐way street (“out of” the QTP). However, immigration into the QTP region has so far received only little attention, despite its potential to shape faunal and floral communities of the QTP. In this study, we investigated centers of origin and dispersal routes between the QTP, its forested margins and adjacent regions for five clades of alpine and montane birds of the passerine superfamily Passeroidea. We performed an ancestral area reconstruction using BioGeoBEARS and inferred a time‐calibrated backbone phylogeny for 279 taxa of Passeroidea. The oldest endemic species of the QTP was dated to the early Miocene (ca. 20 Ma). Several additional QTP endemics evolved in the mid to late Miocene (12–7 Ma). The inferred centers of origin and diversification for some of our target clades matched the “out of Tibet hypothesis’ or the “out of Himalayas hypothesis” for others they matched the “into Tibet hypothesis.” Three radiations included multiple independent Pleistocene colonization events to regions as distant as the Western Palearctic and the Nearctic. We conclude that faunal exchange between the QTP and adjacent regions was bidirectional through time, and the QTP region has thus harbored both centers of diversification and centers of immigration.

## Graphical abstract

The Qinghai‐Tibet Plateau (QTP) is considered a source region for colonization of adjacent areas such as the northern Palearctic. Using an ancestral area reconstruction approach on a phylogeny of 279 bird species, we suggest that faunal exchange between the QTP and adjacent regions was bidirectional through time and the QTP region has harbored both centers of diversification (for example of snowfinches) and centers of immigration.

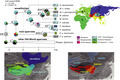

## 1 INTRODUCTION

**Figure 1:**
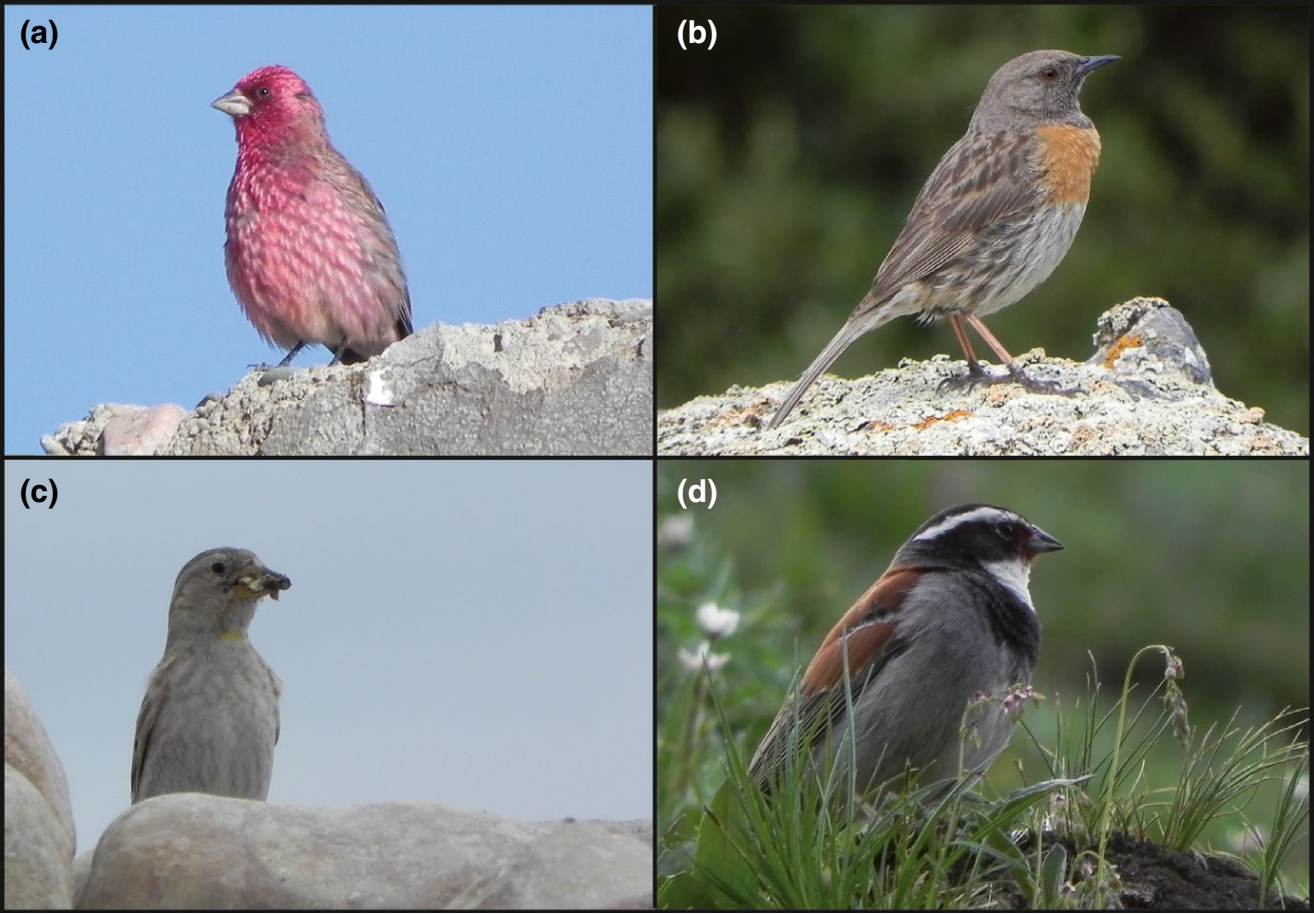
Passerine bird species of the Qinghai‐Tibet Plateau (QTP); QTP endemics, wide distribution range on the plateau: (a) streaked rosefinch, *Carpodacus rubicilloides*; (b) robin accentor, *Prunella rubeculoides*; (c) rock sparrow, *Petronia petronia*, widespread trans‐Palearctic distribution; (d) Tibetan bunting, *Emberiza koslowi*, narrow‐range endemic of the QTP; all photos: M.P., Qinghai, China, June 2013

Many of the World's biodiversity hotspots are located in large mountain systems, such as the Andes, the East African Arc or the Himalayas (Marchese, 2015) and the role of mountains in organismic evolutionary diversification is considered to be manifold (Fjeldså, Bowie, & Rahbeck, 2012; Hoorn, Perrigo, & Antonelli, 2018; Muellner‐Riehl, 2019; Rahbek, Borregaard, Antonelli, et al., 2019; Rahbek, Borregaard, Colwell, et al., 2019). On the Eurasian continent, the Qinghai‐Tibet Plateau (QTP) and its flanking mountain systems constitute the largest and probably most diverse area of montane species richness (Aliabadian, Sluys, Roselaar, & Nijman, 2008; Fjeldså et al., 2012). For example, passerine bird diversity follows a gradient from warm and humid forest ecosystems harboring 358 species in the Eastern Himalayas (Price et al., 2014) and 441 in northern Myanmar (Renner et al., 2015) toward less diverse avian communities in the colder and drier Western Himalayas (Price et al., 2011). Alpine avian communities on the QTP are generally less species rich than those of the Himalayan forest ecosystems (Fjeldså et al., 2012), however, they harbor a couple of wide‐range and narrow‐range endemics as well as widespread trans‐Palearctic species (Figure 1).

**Figure 2:**
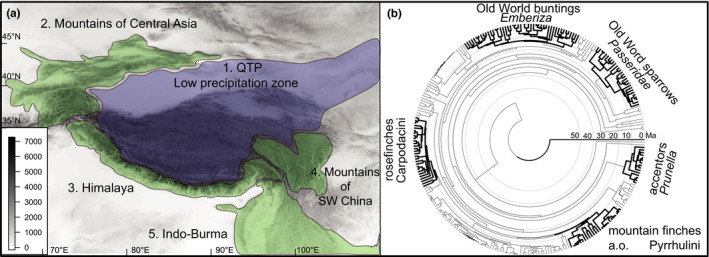
(a) Target region, the Qinghai‐Tibet Plateau (area 1) and the biodiversity hotspots along its forested margins (areas 2–5), modified from Favre et al. (2015); (b) phylogeny for 279 species of Passeroidea, five target clades representing five independent radiations involving QTP species marked in bold

The three major hotspots of diversity at the QTP fringes are the Mountains of Central Asia in the West, the Himalayas in the South, and the Mountains of Southwest China (i.e., the Hengduanshan and its northward extensions) in the East (Figure 2a; Favre et al., 2015). As the largest and highest plateau on Earth, the QTP extends across a surface area of about 2.3 million km^2^, comprising the largest continuous area of montane grasslands of the World (Olson et al., 2001). The alpine environments of the QTP and its flanking mountain systems have been declared as the “Third Pole” (Yao et al., 2012). As such, the QTP recently became famous as the source area of cold‐adapted organisms of the Eurasian fauna (e.g., the Pleistocene mammal fauna: Wang et al., 2015) or the Holarctic alpine flora (Favre et al., 2016). However, the “out of Tibet hypothesis” for the montane and alpine fauna and flora of Eurasia is not a particularly new concept, dating back to the work of the German ornithologist Hugo Weigold in the early 20th century. He was the first to postulate a Tibetan center of diversification (“Entwicklungszentrum”) for Palearctic terrestrial vertebrates, and earliest emergences of Tibetan faunal elements already during the Early Tertiary (Weigold, 1935, 1949, 2005). Weigold's ideas fell into oblivion, until the out of Tibet hypothesis was reanimated in the discussion on the Tibetan origin of Palearctic cold‐adapted mammals inferred from fossil evidence (Deng et al., 2011; Tseng, Li, & Wang, 2013; Tseng et al., 2014; Wang, Li, & Takeuchi, 2016; Wang, Tseng, Li, Takeuchi, & Xie, 2014). Some of these studies suggested a “Himalayan origin” (Wang et al., 2014) or “Central Asian origin” (Tseng et al., 2013) of their study organisms, but all these examples were later reviewed in the context of an “out of Tibet” colonization of the adjacent regions in the North (e.g., Qiu, 2014; Wang et al., 2015). Besides this terminological inaccuracy, the emerging view from analysis of the QTP fossil record was therefore a “one‐way view” of faunal interchange between the QTP (including its margins; Figure 2a) and adjacent bioregions, excluding any reverse movement into Tibet (Tseng et al., 2014; Wang et al., 2014, 2016).

Recent phylogenetic studies have suggested or presumed a one‐directional “out of Tibet” or a “out of Himalayas” dispersal pathway, too, but these were typically focusing on single species or a single, species‐poor clade (e.g., Fuentes‐Hurtado, Hof, & Jansson, 2016; Liu et al., 2017; Voelker, Semenov, Fadeev, Blick, & Drovetski, 2015; and other examples in Table S1). However, work on a larger taxonomic group (e.g., speciose alpine taxa like *Saxifraga*, Ebersbach et al., 2017) suggested that both in situ speciation and immigration played a key role in the evolution of alpine faunal and floral assemblages of the QTP (e.g., Hauenschild et al., 2017) or of the Himalayas (Johansson et al., 2007; compare Table S1).

**Figure 3:**
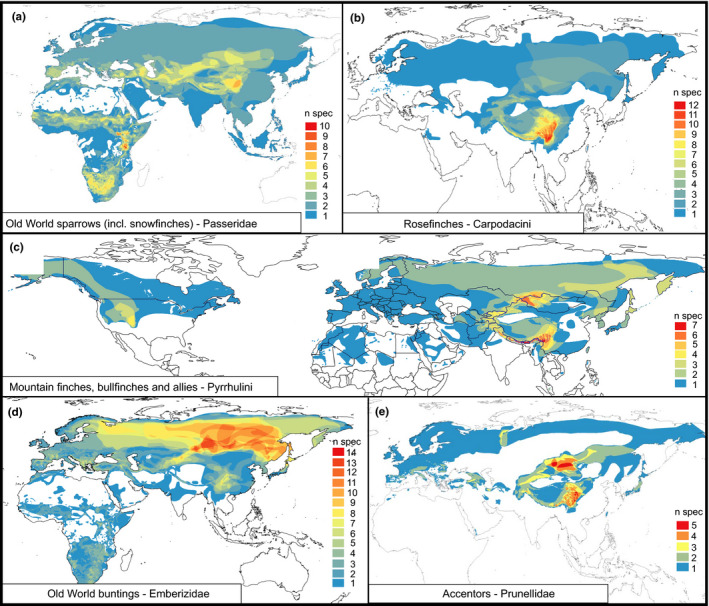
Spatial distribution of species richness and diversity hotspots for all five target clades of Passeroidea; diversity heat maps were compiled with QGIS using shape files inferred from BirdLife International and NatureServe (2015) and from the IUCN Red List (2019); colors indicate regional species richness (high = red; low = dark blue)

In this study, we compare patterns of biogeographic history among five subclades of Passeroidea, a superfamily of passerine birds (Johansson, Fjeldså, & Bowie, 2008; Selvatti, Gonzaga, & de Morales Russo, 2015). These subclades represent five independent passerine radiations (Figure 2b), representing ideal model groups for the study of biogeographic history of QTP faunal assemblages: the studied species are montane and alpine birds distributed across all mountain systems of the Holarctic (Figure 3) and include 29 endemics to the QTP region. For these five passerine clades, biogeographic analyses and ancestral area reconstructions are either missing, incomplete, or provide contradictory results (Drovetski et al., 2013; Liu et al., 2017; Tietze, Päckert, Martens, Lehmann, & Sun, 2013).

Our five studied passerine clades encompass characteristic faunal elements of the QTP region and include several lineages considered as flagship species by Weigold (2005) for the high alpine grasslands and rocky hillslopes of the QTP: (a) Old World sparrows (Passeridae) comprising snowfinches (*Montifringilla* and allies; two genera *Onychostruthus* and *Pyrgilauda* are endemic to this region). The area of high Passeridae species richness at the eastern margin of the QTP is certainly due to the high snowfinch diversity in this region (Figure 3a; a second hotspot in East Africa is characterized by a high diversity of *Passer* species), (b) the mountain finches (genus *Leucosticte*), with three species in the QTP region and further three in the Nearctic. This genus along with bullfinches and other allies belong to tribe Pyrrhulini (clade 6 in Zuccon, Prŷs‐Jones, Rasmussen, & Ericson, 2012). With another group, (c) the rosefinches (Carpodacini: clade 5 in Zuccon et al., 2012), both tribes of finches (Fringillidae) have areas of highest species richness in the Sinohimalayas (Pyrrhulini also north of the QTP; Figure 3b,c). Weigold (2005) hypothesized that all extant rosefinches originated from a Tibetan ancestor but later diversified in peripheral refuges at the southern and the eastern QTP margin when forest habitats vanished from the central plateau region. Furthermore, we investigate (d) Old World buntings (Emberzidae). Although the hotspot of highest bunting species richness is located in the Eastern Palearctic, a few taxa form a secondary hotspot of species richness at the eastern QTP margin (Figure 3d). According to Weigold (2005) the endemic Tibetan bunting, *Emberiza koslowi* (Figure 1d) represents an “autochtonous faunal element” of the QTP (i.e., he assumed that this species originated in situ on the QTP), whereas the crested bunting (*Emberiza lathami*) had originated from a tropical ancestor southwest of the QTP. Finally, (e) accentors, in the Palearctic family Prunellidae, are most speciose at the northeastern and southeastern QTP margin (Figure 3e).

Our five target clades encompassed 132 Holarctic species, including 29 endemics of the QTP and its forested margins (compare “Material and Methods,” “Species distributions”). Assuming an in situ origin of all QTP endemics that was associated with the supposedly recent formation of the plateau region (Liu, Gao, Chen, & Lu, 2002 and references therein), recent orogenetic processes should have promoted the rise of species‐level lineages endemic to the QTP from the Middle Miocene on (Shang et al., 2015). However, such a tight coupling of young evolutionary events (species splits, adaptive radiations in the QTP region) with orogenetic post‐Miocene events (“young Tibet hypothesis”) has recently been critically challenged by Renner (2016). Indeed, there is evidence of ancient QTP species that evolved prior to the Miocene (Baker, Pereira, & Paton, 2007; Päckert, Sun, et al., 2015). Furthermore, some ancient Tibetan and Himalayan faunal elements already emerged during the Eocene and later diversified in situ on the QTP over a long period of time (Martens, 2015; Mosbrugger, Favre, Muellner‐Riehl, Päckert, & Mulch, 2018 and references therein). These findings are in accordance with Weigold’s (2005) theory of an early Tertiary origin of the endemic fauna of Tibet, though he was originally driven by the idea that diversification of the Tibetan fauna was firmly associated with the uplift of the QTP.

In accordance with Weigold's theories and with more complex biogeographic patterns derived from large phylogenies and comparison across clades, we searched for evidence of ancestral areas of origin for our target groups on the QTP itself and along its margins (Figure 2a), and also in adjacent regions beyond the QTP region. We therefore provide one of very few case studies that compare patterns of biogeographical history across different clades from a speciose monophyletic group of organisms.

## 2 MATERIALS AND METHODS

### 2.1 Sampling strategy

Biogeographic inferences for our five target clades needed to be anchored within a broader biogeographic scenario, including a broad selection of closely related as well as distant clades. We therefore assembled a DNA sequence dataset for Passeroidea, a highly speciose crown clade of Passeriformes that comprises 25 avian families (see Figure S1; Table S2). For all our target clades, our taxon sampling was 100% or near 100% (missing only *Montifringilla theresae* from Central Asia and *Carpodacus sillemi* from the QTP). We furthermore included a few subspecific taxa that are currently not accepted as “good species” but nevertheless represent distinct resident lineages (Table S2). These subspecies‐level taxa represented vicariants occurring in different regions of a species’ range (in Eurasia our areas B–H) and in some species, distinct subspecific lineages correspond to distinct phenotypes, like in the common bullfinch (*Pyrrhula pyrrhula*: Töpfer et al., 2010). Divergence times between all these subspecific taxa equal or even exceed the minimum divergence times estimated for a good species pair of the target clade they are nested in. These species pairs are as follows: (a) for snowfinches *Pyrgilauda davidiana*/*P. blanfordi* (Delgado, Bettega, Martens, & Päckert, 2019); (b) for Carpodacini *Carpodacus rubicilloides* (Figure 1a)/*C. rubicilla* and *C. thura*/*C. dubius* (Tietze et al., 2013); (c) for Pyrrhulini *Leucosticte tephrocotis*/*L. australis*/*L. atrata* (Drovetski, Zink, & Mode, 2009); (d) for buntings: *Emberiza hortulana*/*E. caesia* and *E. citrinella*/*E. leucocephalos* (Päckert, Sun, et al., 2015); (e) Prunellidae: *Prunella ocularis*/*P. fagani* (Liu et al., 2017).

### 2.2 DNA extraction, PCR, and sequencing

To complete taxon sampling and coverage of loci for our target clades, we used samples and DNA extracts available from previous analyses (Päckert, Sun, et al., 2015; Päckert, Martens, Sun, & Strutzenberger, 2016; Tietze et al., 2013; Töpfer et al., 2011; for newly generated sequences see Table S2). We extracted DNA from new samples using Qiagen blood and tissue kits according to the manufacturer's instructions. Our sequence data set included four loci: the mitochondrial cytochrome‐*b* (cyt*b*) and NADH dehydrogenase subunit 2 (ND2), as well as the nuclear introns ornithine decarboxylase (ODC) intron 7, and myoglobin (myo) intron 2. Overall, we used general and previously published primers (for PCR and sequencing primer combinations and PCR settings see Table S3). However, we utilized taxon‐specific primer pairs for PCR and sequencing of ND2 for Passeridae by Belkacem et al. (2016) and of ODC for rosefinch and bunting samples (Table S3). We purified PCR products using ExoSap‐IT (GE Healthcare; adding 0.1 ml ExoSap‐IT solution in 4 ml H2O to each sample; PCR settings: 37°C for 30 min, 94°C for 15 min) and sequenced the purified PCR products, which was performed with BigDyeTM 3.1 Dye Terminator Cycle Sequencing Kits (Applied Biosystems), according to the manufacturers’ instructions. Sequencing products were purified by salt/ethanol precipitation or by using Sephadex (GE Healthcare), and sequenced in both directions on an ABI 3130xl DNA sequencer.

### 2.3 Data assembly

Sequences for core target taxa were compiled from previous studies (*n* = 101 species analyzed in Päckert et al., 2016; further taxa added from Tietze et al., 2013 and Päckert, Sun, et al., 2015). We added missing species and filled gaps in sequence coverage with newly generated sequences. To have all families of Passeroidea represented by at least one species, we included further sequences from GenBank. This is in accordance with Cai et al. (2018) who emphasized that estimates of ancestral range for ingroups (i.e., our target clades) are strongly affected by the ranges of outgroups (i.e., sister clades of our target clades). We added two passerine outgroup species: *Bombycilla garrulus* (Bombycilloidea as the sister clade of Passerida) and *Acanthisitta chloris* (Acanthisittidae as the earliest offshoot of the Passeriformes) and three further nonpasserine outgroup taxa (Table S2). Newly generated sequences were joined with sequences obtained from previous studies and sorted using the R package “ape” (Paradis, Claude, & Strimmer, 2004; Paradis & Schliep, 2019). Sequences were assembled using custom scrips in R (R Development Core Team, 2016) and aligned with the stand‐alone version of MAFFT 7.273 (Katoh & Standley, 2013) with automatic selection of the appropriate alignment strategy, the scoring matrix set to “200PAM/*κ* = 2,” and the gap opening penalty set to 1.53 (with a gap extension penalty of 0.123). The obtained sequence alignments were manually checked for errors. The total sequence alignment had a length of 4,813 bp and totaled 281 taxa.

All newly generated sequences have been deposited at GenBank under the following accession numbers: MT210104–M210119 (cyt*b*), MT210120–MT210147 (ND2), MT277429–MT277443 (myo), and MT336176–MT336215 (ODC).

### 2.4 Phylogenetic reconstruction and divergence‐time estimation

Phylogenetic inference and divergence‐time estimation were performed with BEAST v1.8.2 (Drummond, Suchard, Xie, & Rambaut, 2012). We partitioned our dataset in accordance with the best‐fitting partitioning scheme that resulted from PartitionFinder v1.1.1 (Lanfear, Calcott, Ho, & Guindon, 2012) with all site models unlinked and one clock model for each gene resulting in a total of eight sites and four clock models. The search for the best strategy relied on the “beast” model‐set and heuristic search. We linked all tree models to one tree model and a birth–death tree prior was applied. As initial condition for the BEAST run, we supplied a starting tree calculated with RAxML v8.2.0 (Stamatakis, 2014). Search for the best‐known likelihood tree was performed with 100 replicates, bootstrap values obtained from a thorough bootstrap run using the autoMRE option (Pattengale, Alipour, Bininda‐Emonds, More, & Stamatakis, 2010) were annotated onto the best‐known likelihood tree. All analyses were performed using the same partitioning scheme as applied for BEAST analyses with the GTRGAMMA model applied to all partitions. The BEAST run was performed using the BEAGLE v2.3 library with a chain length of 1.1 × 108 generations with trees being sampled every 10,000 generations. The chain was inspected for convergence and sufficient ESS values (>200) with Tracer v1.6 (Rambaut & Drummond, 2007). Trees were summarized with TreeAnnotator v1.8.2, where median heights were annotated to the maximum clade credibility (MCC) tree.

We used eight calibration points in order to obtain estimates for node ages (modified from Päckert et al., 2016; see Table S4). Additionally, we applied a normal prior to the root age to avoid the occurrence of implausibly old root ages. Instead of using hard boundaries, we adopted a “soft boundary” strategy where the desired calibration is achieved by setting priors in such a way that 97.5% of the probability density lie within the desired minimum and maximum boundaries (Benton, Donoghue, & Asher, 2009). All calibrated nodes were forced to be monophyletic. Sequence alignments and tree files are deposited at Dryad under https://doi.org/10.5061/dryad.xksn02vd0.

### 2.5 Species distributions

**Figure 4:**
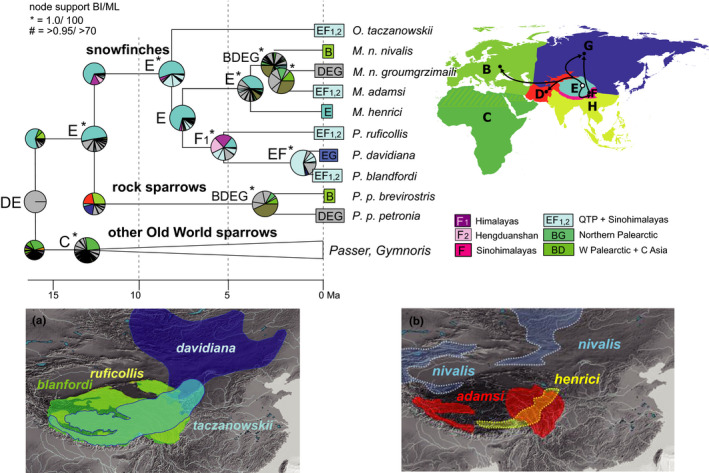
“Out of Tibet” dispersal of snowfinches (*Montifringilla*, *Onychostruthus*, *Pyrgilauda*) and rock sparrows (*Petronia*), only the respective clade of the time‐calibrated Passeroidea MCC tree is shown; node support from Bayesian inference of phylogeny (BI) and Maximum Likelihood (ML) indicated by symbols explained above the tree (no symbol for support values below 0.95/80); biogeographical reconstruction based on a dispersal–extinction–cladogenesis (DEC) model; letters at nodes show best states, pie charts show per‐area probabilities inferred from ARR2 (nine areas); main dispersal events sketched on the map including area codes (right); color codes for most frequent ancestral area combinations below map (right); extant patterns of sympatry on the QTP shown on maps below for (a) small snowfinches, three *Pyrgilauda* species and one *Onychostruthus* species (BirdLife International and NatureServe, 2015); (b) large species of *Montifringilla* (Asian distributions of *M. nivalis* and *M. henrici* according to Gebauer et al., 2006)

**Figure 5:**
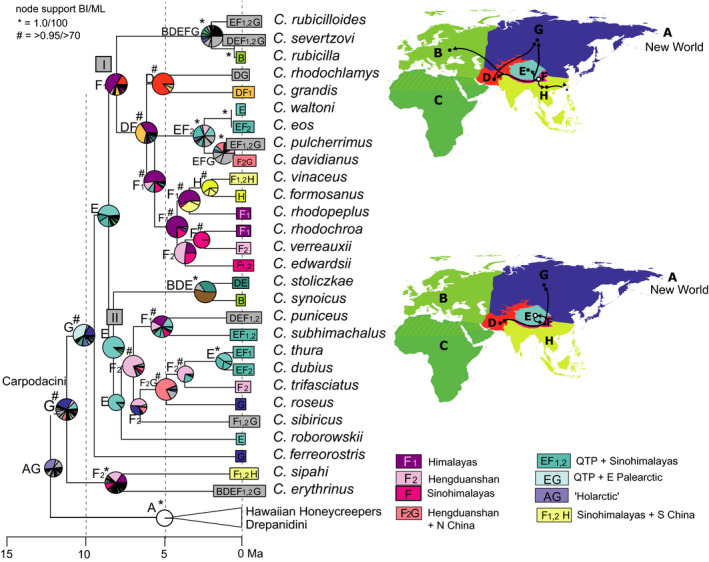
“Out of Himalaya” dispersal of rosefinches (*Carpodacus*; only the respective clade of the time‐calibrated Passeroidea MCC tree is shown); node support from Bayesian inference of phylogeny (BI) and Maximum Likelihood (ML) indicated by symbols explained above the tree (no symbol for support values below 0.95/80); biogeographical reconstruction based on a dispersal–extinction–cladogenesis model (DEC); letters at nodes show best states, pie charts show per‐area probabilities inferred from ARR2 (nine areas); main dispersal events sketched on the map including area codes (right); color codes for most frequent ancestral area combinations below map (right)

**Figure 6:**
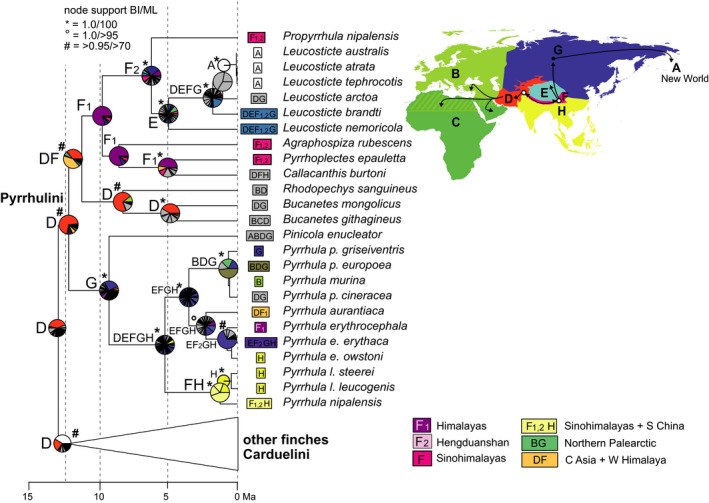
“Out of Himalaya” dispersal of mountain finches (*Leucosticte*) and allies (only the respective clade of the time‐calibrated Passeroidea MCC tree is shown); node support from Bayesian inference of phylogeny (BI) and Maximum Likelihood (ML) indicated by symbols explained above the tree (no symbol for support values below 0.95/80); biogeographical reconstruction based on a dispersal–extinction–cladogenesis model (DEC); letters at nodes show best states, pie charts show per‐area probabilities inferred from ARR2 (nine areas); main dispersal events sketched on the map including area codes (right); color codes for most frequent ancestral area combinations below map (right)

**Figure 7:**
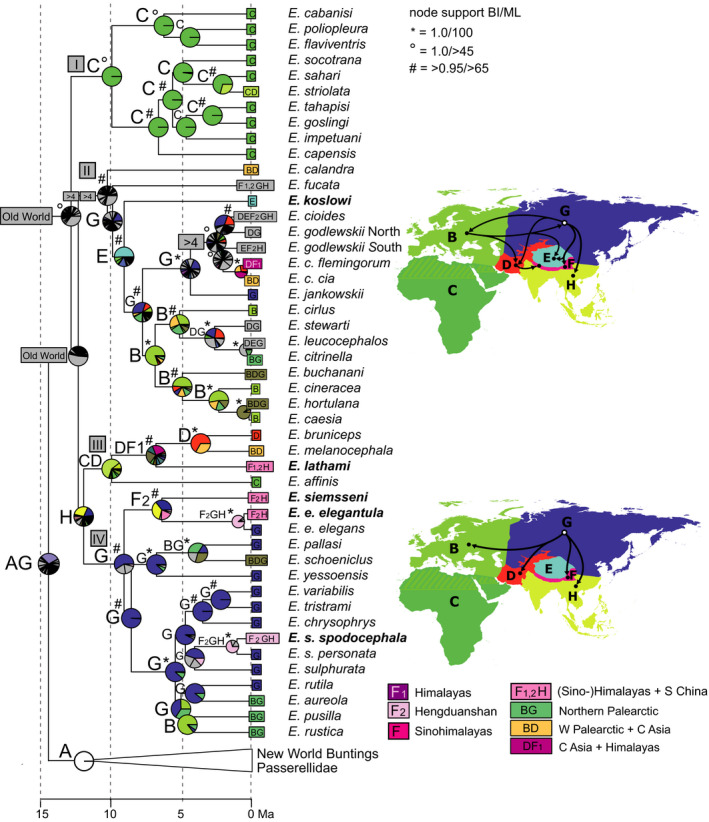
“Into Tibet” dispersal of Old World buntings (*Emberiza*, four clades I‐IV indicated in gray boxes; only the respective clade of the time‐calibrated Passeroidea MCC tree is shown); node support from Bayesian inference of phylogeny (BI) and Maximum Likelihood (ML) indicated by symbols explained above the tree (no symbol for support values below 0.95/80); biogeographical reconstruction based on a dispersal–extinction–cladogenesis model (DEC); letters at nodes show best states, pie charts show per‐area probabilities inferred from ARR2 (nine areas); main dispersal events sketched on the map including area codes (right); color codes for most frequent ancestral area combinations below map (right)

As basis of our biogeographic analysis, we delineated eight regions (see maps in Figures 3, 4, 5, 6, 7 with the New World as region A). Those areas, located outside the QTP region, were designed to loosely follow the classic biogeographic realms and zoogeographical boundaries (Ficetola, Mazel, & Thuiller, 2017), as done in other studies (e.g., Favre et al., 2016). We divided our target region into the high alpine Tibetan plateau (area E; Figure 2a: region 1) and two major flanking mountain systems: (a) the Central Asian Mountains in the West (area D; Figure 2a: region 2) and (b) the Sinohimalayas comprising the Himalayas (Figure 2a: region 3) and the Hengduanshan (Figure 2a: region 4) in the South and Southeast (area F). The Sinohimalayas represent one of three major global hotspots of avian diversity (e.g., Cai et al., 2018) with a high richness of both ancient and recent species (together with the Andes and the mountains of the African Arc; Fjeldså et al., 2012). Among 29 species endemic to the QTP region, ten species were present in only one area: four on the Tibetan Plateau [Figure 2a: area 1], three in the Himalayas [Figure 2a: area 3], two in the Hengduanshan [Figure 2a: area 4] and one in the Central Asian Mountains [Figure 2a: area 2]. Furthermore, our target groups comprise nineteen endemic species present in two or more areas of the QTP region (Figure 2a), among them four species which are endemic to the Sinohimalayas.

We used QGIS v2.4 (QGIS Development Team, 2014) to draw shapefiles representing the maximum nine geographic regions in our analysis. We established a workflow for automated extraction of bird distributional ranges, using bird distribution data provided by BirdLife International and NatureServe (2015) in ESRI shapefile format. Geographic ranges encoded in BirdLife International (BLI) shapefiles were filtered to only represent year‐round and breeding ranges of natively occurring species according to three types of distribution encoded in the attribute table of a shapefile. A species’ range was therefore reduced to those polygons corresponding to the following categories: type presence: extant; type origin: native; type seasonality: resident and breeding season. Subsequently, a dissolve operation was applied in QGIS to join all separate features for each species resulting in each species being represented by a single polygon feature. The intersection function (QGIS) was then used to obtain a list of all intersections between the geographic area polygons and the bird distribution polygons. The resulting list was converted to a matrix using the “xtabs” function (Rbase) and the “as.matrix.xtabs” command (DescTools).

In a second step, we made manual corrections to the area matrix for a couple of reasons. First, our data set included two species recognized by Clements et al. (2015) but not by BLI (i.e., their distributions were included in shape files of other species). These were as follows: *Montifringilla henrici* [listed as subspecies of *M. nivalis* by BLI; distribution corrected according to Gebauer, Eck, Kaiser, Lei, and Martens (2006) and Martens and Eck (2003) see Figure 4b]; *C. formosanus* [listed as ssp. of *C. vinaceus* by BLI]; this species occurs only on Taiwan [included in our area H]; for distinctiveness of *C. formosanus* according to an integrative taxonomic approach compare Wu et al., 2011). Second, based on modeled species distributions Ramesh, Gopalakrishna, Barve, and Melnick (2017) has raised the concern that for narrow‐range endemics, BirdLife International shape files might overestimate the true extent of distribution range. However, their study was limited to the regional avifauna of the Western Ghats and we consider these deviations between projected BLI distributions and modeled distributions (Ramesh et al., 2017) of minor importance for our transcontinental study (even our two smallest areas the Himalayas and the Hengduanshan are 1.7–3.7 times larger than the Western Ghats). Furthermore, other studies suggested a good accordance of potential distribution models (PDMs) and BLI distributions for several Mexican bird species (Ortega‐Huerta & Vega‐Rivera, 2017). Nevertheless, we took into account that automatic extraction of species ranges from BLI shape files might cause false‐positive occurrence of a species in an area with only marginal overlap at the very margins of its breeding range (examples of *Carpodacus* rosefinches are shown in Figure S2). Because we consider this a potential bias of our automatic extraction of bird ranges from BirdLife shapefiles, we deleted these marginal areas from the area matrix. We cross‐checked our area classification with published range maps in the Atlas of Palearctic Breeding Birds (for example for rosefinches, Carpodacini, in Martens & Sun, 2008) as was done in previous studies (e.g., Drovetski et al., 2013) (further atlas volumes consulted: Dathe & Loskot, 1992; Stresemann & Portenko, 1960, 1976, 1977, 1978, 1980, 1981; Stresemann, Portenko, & Mauersberger, 1971; Stresemann, Portenko, Dathe, & Mauersberger, 1974). Our final area matrix is included in our data package deposited at Dryad under https://doi.org/10.5061/dryad.xksn02vd0.

### 2.6 Ancestral‐range reconstruction (ARR)

We used R package BioGeoBEARS (Matzke, 2013) for ancestral‐range reconstruction (ARR). The maximum clade credibility tree obtained from BEAST was used as input tree (we kept the waxwing, *B. garrulus*, as the closest outgroup of Passeroidea and pruned all further outgroups used for fossil calibration from the input tree shown in Figure S1). We fitted the dispersal, extinction, and cladogenesis (DEC) model to the time‐calibrated Passeroidea tree (for further details, see Appendix S5). We used a “dispersal multipliers” matrix, allowing dispersal between all areas, favoring adjacent ones (1.0), but penalizing slightly (0.5) dispersal between nonadjacent areas interconnected by a third one (in a land continuum), and penalizing strongly (0.01) long distance dispersal between very distant continents (e.g., Europe and the New World; see Tables S5a,b).

We performed two alternative runs with BioGeoBEARS: one with the Sinohimalayas classified as a single area F (ARR1; Figure S3) and a second run with the Himalayas and the Hengduanshan as two separate areas (F_1_, F_2_; ARR2) with the Mekong‐Salween divide as one of the main geographic barriers (compare Liu et al., 2016; Päckert et al., 2012). Separate treatment of these two mountain regions also takes into consideration the different orogenetic histories and the younger age of the Hengduanshan (Favre et al., 2015; and references therein). Dispersal multipliers were adjusted to the latter analyses with eight and nine areas, respectively (Tables S5a,b). Our final dispersal multiplier matrices were included in our data package deposited at Dryad under https://doi.org/10.5061/dryad.xksn02vd0.

## 3 RESULTS

### 3.1 Phylogeny and origin of Passeroidea

Phylogenetic analyses with BEAST and RAxML resulted in largely congruent topologies for the major families of Passeroidea (Figure S1; autoMRE bootstrap converged after 200 replicates). Among all Passeroidea, Przevalski's finch, *Urocynchramus pylzowi*, represented the oldest species‐level lineage endemic to the QTP (Figure S3). The split from its sister group (Ploceidae) was dated to a mean age of 20 Ma [16.4–24.7 Ma], which corresponds to the onset of the diversification for all our five target clades (see below). All fourteen endemic species of the QTP region (that occurred in a single area, including four Sinohimalayan endemics) emerged during the late Miocene (e.g., Tibetan bunting, *E. koslowi*) or early Pleistocene (e.g., Tibetan Snowfinch, *M. henrici*).

In ARR1 and ARR2, area uncertainty was high at the basal nodes of the Passeroidea tree, however, best states suggested a Holarctic ancestral range of Passeroidea (Afrotropics and Oriental region were not included in the ancestral range; Figure S3). In contrast, for several suprageneric clades, ancestral ranges were limited to one region only, for example, a Nearctic origin of “Emberizoidea” sensu Barker, Burns, Klicka, Lanyon, and Lovette (2013) (see Figure S1) and Fringillidae (see Figure S3).

### 3.2 Biogeographical history of montane and alpine target groups of Passeroidea

We found contrasting areas of diversification and dispersal routes between the QTP and adjacent regions for our five target clades. Generally, there was large congruence between reconstructions based on eight areas (ARR1) and nine areas (ARR2), respectively. Separate classification of the Himalayas and the Hengduanshan as two areas F_1_ and F_2_ (ARR2) often resulted in ancestral ranges with highest per‐area probabilities in either of the two regions (at nodes with an ancestral range in the Sinohimalayas in ARR1; Figures S3 and S4). In the following, we present and discuss the results from ARR2 based on nine areas and refer to deviations among ARRs only in the one case that showed an effect of the total number of areas on area uncertainty.

A QTP origin (area E) was recovered for snowfinches and rock sparrows (Figure 4), with a mean root age estimate of 12.4 [9.9–15.3] Ma for the onset of their radiation. For their sister clade (Old World sparrows of genera *Passer* and *Gymnoris*) an African area of origin was estimated (best state and highest per‐area probabilities). Genus *Passer* includes several widespread species whose ranges extend into the QTP region (Figures S3 and S4), however, none of these would represent a QTP endemic that had colonized the QTP from elsewhere. Two main clades of snowfinches (*Pyrgilauda*, small species; *Montifringilla*, large species) separated at about 7.6 [6.0–9.6] Ma. Area E was the best state and had highest per‐area probabilities at most nodes of the snowfinch clade. Only for the ancestral range of small *Pyrgilauda* species, the Sinohimalayas had the highest per‐area probabilities in ARR2 (two areas F_1_, F_2_, whereas from ARR1 again area E resulted as best state for the ancestral range of *Pyrgilauda*). The *Montifringilla* clade included the only snowfinch species that during the late Pleistocene dispersed as far as to the Western Palearctic (*M. nivalis*; Figure 4).

Areas of origin and diversification in the QTP region were also reconstructed for both our target clades of finches (Fringillidae). The onset of rosefinch (Carpodacini) radiation was dated to 11.2 [9.5–13.3] Ma and involved major centers of diversification on the QTP (area E) and in the Sinohimalayas (areas F_1_ and F_2_; Figure 5). Their sister clade, the Hawaiian Honeycreepers (Drepanidini), represents a solely Nearctic group. Two larger rosefinch clades diversified in different areas of the QTP region: Clade I comprised 15 species that diversified in the Himalayas (highest or high per‐area probabilities for area F_1_ at five nodes; Sinohimalayas best state; Figure 5, Figure S3). The Himalayan rosefinch radiation included one early dispersal event to Central Asia (area D) and a more recent dispersal event to southern China and Taiwan (area H; Figure 5). For the second major rosefinch clade II, comprising ten species, area E had the highest per‐area probabilities at the two successively basal nodes (Figure 5). However, at several nodes of that second clade, the Hengduanshan (area F_2_) had highest (or high) per‐area probabilities or was recovered as best state (Figure 5). Both groups included a terminal Pleistocene dispersal event to the Western Palearctic (area B; Figure 5: clade I, *C. rubicilla*; clade II: *C. synoicus*).

A Himalayan origin was reconstructed for another finch clade, Pyrrhulini, that comprised the mountain finches of genus *Leucosticte*, bullfinches of genus *Pyrrhula* and allies from five other genera (Figure 6; highest per‐area probabilities for area F_1_, and best state F_1_ at several clades). Pyrrhulini are sister to a diverse group of finches (tribe Carduelini) that among others includes several subclades restricted to the New World or to the Afrotropics (Figure S3). The high per‐area probability for a New World origin of Carduelini (Figure 6) is certainly due to the basal split leading to the Nearctic house finch clade (genus *Haemorhous*; Figure S3). One Tibeto‐Himalayan endemic, the Tibetan serin (*Serinus thibetanus*), emerged from the Carduelini clade, sister to a New World clade of siskins (Figure S3: the Eurasian siskin, *Spinus spinus*, belongs to this clade and has very likely colonized the Palearctic from the New World). Strikingly, this Tibeto‐Himalayan endemic does not have any close phylogenetic relationship with other QTP species. The onset of Pyrrhulini diversification was dated to a mean age of 12.3 [10.2–14.4] Ma and their radiation also included a very recent terminal dispersal event of mountain finches (*Leucosticte*) into the Nearctic (area A_1_; Figure 6). The entire Himalayan finch group belonged to a larger finch clade, for which a Central Asian ancestral range was inferred (highest per‐area probabilities for area D or DF).

In the buntings (*Emberiza*) several extant taxa from the QTP were nested within two larger clades that did not originate and diversify in the QTP region. Their sister group is represents a New World clade, the Passerellidae (Figure 7). An Eastern Palearctic center of origin and diversification was unanimously assigned to a clade comprising sixteen species including the Chinese endemic *E. siemsseni* and further distinct subspecific lineages of East Asian species (Figure 7, clade IV). Breeding ranges of all QTP taxa of clade IV are mainly restricted to the Hengduanshan (area F_2_ and parts of the adjacent QTP, area E; Figure 7). The ancestral area of clade II was equivocal (many combinations with low per‐area probabilities at the two successively basal nodes; Figure 7). The Tibetan bunting (*E. koslowi*) was one of three early offshoots of clade II (mean split age 9.1 [7.5–10.9] Ma) and the QTP (area E) was recovered as the ancestral range at that node (highest per‐area probabilities and best state; Figure 7).

**Figure 8:**
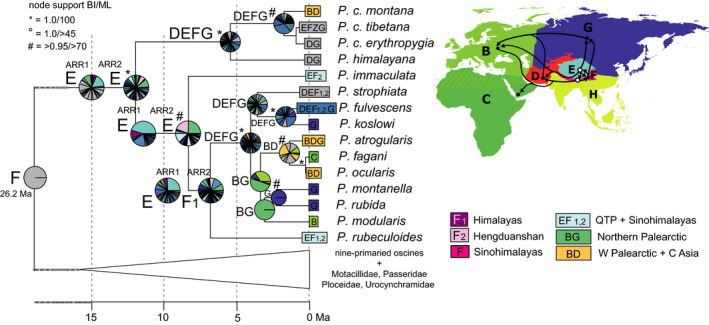
“Out of Tibet” dispersal of accentors (*Prunella*; only the respective clade of the time‐calibrated Passeroidea MCC tree is shown); node support from Bayesian inference of phylogeny (BI) and Maximum Likelihood (ML) indicated by symbols explained above the tree (no symbol for support values below 0.95/80); biogeographical reconstruction based on a dispersal–extinction–cladogenesis model (DEC); letters at nodes show best states, pie charts show per‐area probabilities inferred from ARR2 (nine areas; plus those from ARR1 with eight areas and less uncertainty at the three basal nodes); main dispersal events sketched on the map including area codes (right); color codes for most frequent ancestral area combinations below map (right)

The onset of accentor (*Prunella*) radiation was dated to 11.9 [9.4–14.7] Ma. The area origin of accentors (*Prunella*) was highly equivocal (low per‐area probabilities for any combination of regions); however, area E (high QTP) was recovered as best state in both ARRs (Figure 8). For the basal nodes, there was less area uncertainty, when the Sinohimalayas were coded as a single area (a total of eight areas in ARR1 instead of nine in ARR2). According to ARR2, the QTP (area E) was the ancestral range of the accentor crown clade with highest per‐area probabilities (Figure 8).

## 4 DISCUSSION

### 4.1 Phylogeny and biogeographic history of Passeroidea

Our Passeroidea tree is in good accordance with previously published phylogenies, most of them based on a less dense taxon sampling (Barker et al., 2013; Davis & Page, 2014; Johansson et al., 2008; Jønsson & Fjeldsa, 2006). The timing of lineage splits inferred from our fossil dating approach is in accordance with other dated phylogenies (Barker et al., 2013; Claramunt & Cracraft, 2015; Moyle et al., 2016). Our ancestral‐range reconstructions suggested a Holarctic origin of Passeroidea, whereas previous studies have suggested either a Palearctic origin (Claramunt & Cracraft, 2015) or an Indomalayan origin (Moyle et al., 2016) of this group. The latter difference is striking, as both studies applied the same global area coding. For our data set, uncertainty of ancestral ranges increased with the number of global areas classified (eight and nine) as illustrated for accentors, Prunellidae (Figure 8). Though previous studies on the biogeographical history of this family relied on even fewer areas (four and six), they predicted a rather broad ancestral range across the entire QTP region (Drovetski et al., 2013; Liu et al., 2017). Apart from different reconstruction methods, deviations between our results and those of the two latter studies might be due to different classifications of global areas on the one hand, and different delimitation of species distribution ranges on the other hand (e.g., inclusion or exclusion of the Himalayan nonbreeding range of *Prunella himalayana*). Thus, the outcome of ancestral area reconstructions might not only depend on the models applied (Clark et al., 2008; Pirie, Humphreys, Antonelli, Galley, & Linder, 2012) and different reconstruction methods (Johansson, Nylinder, Ohlson, & Tietze, 2018; Tietze et al., 2013), but also the area‐coding approach. This has to be kept in mind for the discussion of the following evolutionary trajectories that we identified for our five Eurasian montane and alpine target clades.

### 4.2 “Out of QTP”—in situ diversification on the QTP and along its forested fringes

Our results suggested a late Miocene origin (at about 8 Ma) and in situ diversification on the QTP and along its margins for snowfinches and for rosefinches of clade II. At the same time, ancestors of the major accentor clades dispersed “out of a Tibet” (area E) to a wide ancestral range including Central Asia and the Northern Palearctic (areas DEFG). There, ancestral lineages of accentors appear to have diversified from the late Miocene onward. In these three examples, that early phase of their radiation (8–5 Ma) coincides with a period of global climate cooling toward the end of the Miocene (reviews in Favre et al., 2015; Mosbrugger et al., 2018), with an intensification of Asian winter monsoon (Holbourn et al., 2018) and increasing aridification of the QTP (Miao et al., 2019; but compare Nie et al., 2017 for a warmer and wetter Miocene climate in the QTP region). Along with a decrease of birch and oak forests and a continuous change toward steppe vegetation on the northern QTP from 8.5 Ma on (Chen & Yang, 2016; Hui et al., 2011), new semiopen plateau habitats could have represented an evolutionary opportunity for the ancestors of extant QTP rosefinches, snowfinches, and accentors.

Adaptive radiations on the QTP appear to have followed general ecogeographic rules. For example, smaller species of snowfinches (*Pyrgilauda*) diversified at the southern QTP margins, whereas larger species (*Montifringilla*) did so at higher latitudes, in the northern QTP margins. Similar accordance to Bergmann's rule (Bergmann, 1847) was found along a latitudinal gradient on the QTP in small mammals such as pikas (Lin, Ci, Zhang, & Su, 2008) and zokors (Zhang, Nevo, Tang, Su, & Lin, 2012).

In the final Holocene phase of alpine passerine radiations, postglacial range expansion resulted in patterns of wide‐range sympatry on the QTP, where today extant endemics occupy different ecological niches of the alpine meadows and grasslands. Snowfinch species of the QTP belong to two different ecological guilds with different habitat preferences for vertical or horizontal habitat complexity (Li et al., 2018). Similarly, all sympatric accentor (*Prunella*) species of the high alpine habitats on the QTP and its margins differ strikingly from each other in habitat preferences and in morphology (Drovetski et al., 2013; Liu et al., 2017). Only a few snowfinch and rosefinch species have colonized alpine habitats of central and western Palearctic mountain systems according to the “out of Tibet” hypothesis (e.g., *M. nivalis*) during the Pleistocene. Likewise, the (Sino‐)Himalayas have been considered a source area of Holarctic montane organisms (Favre et al., 2016; Matuszak, Muellner‐Riehl, Sun, & Favre, 2016; Pisano et al., 2016; Wen, Zhang, Nie, Zhong, & Sun, 2014) and “out of Himalayas” colonization in our target groups was suggested for the transcontinental colonization of Nearctic mountain systems from a Himalayan area of origin in mountain finches (*Leucosticte*; this study). A single rosefinch species emerged from dispersal out of the Himalayas to a subtropical region, that is, *C. formosanus* on Taiwan. Though none of the five radiations of montane Passeroidea reached into tropical regions (except of one bullfinch species, *Pyrrhula leucogenis*, on the Philippines), other clades of Passeroidea encompass tropical radiations across Southeast Asia (including the Sinohimalayas) such as those of sunbirds and flowerpeckers (Nectariniidae, *Aethopgya*: Hosner, Nyári, & Moyle, 2013; Dicaeidae: Nyári, Peterson, Rice, & Moyle, 2009). The Sinohimalayas were also considered the area of origin and in situ diversification of babblers, a highly diverse passerine group that from the early Miocene on colonized the entire Old World including the Tropics where regions of great babbler diversity can be found today (Cai et al., 2020).

Moreover, biogeographic scenarios reconstructed for Passeroidea did not only include faunal interchange among the QTP and adjacent regions but also within the QTP region itself (e.g., exchange between areas D, E, and F).

### 4.3 Faunal exchange between the plateau region and the QTP fringes

The discussion about processes fostering diversification within the QTP region has often been limited to phylogeographic patterns, that is, intraspecific genetic divergence as a result of Pleistocene range shifts to glacial refuges along its margins and successive Holocene range expansion (e.g., Lei, Qu, & Song, 2014; Yang, Dong, & Lei, 2009). This particular evolutionary scenario was recently summarized in the “contraction/recolonization” hypothesis by Muellner‐Riehl (2019). However, for several of the Tibetan radiations during the Ice Ages also “platform refugia” and “microrefugia” (Muellner‐Riehl, 2019) might explain the current patterns of sympatry and parapatry, such as for rosefinches of the *Carpodacus pulcherrimus* complex (this study, Figure 5; Tietze et al., 2013). Moreover, faunal exchange between the plateau and its peripheral forested mountain regions must have already occurred prior to the Pleistocene. The scenario of in situ diversification in the Himalayas for two finch clades (rosefinches: Figure 5; mountain finches and allies: Figure 6) suggested a major phase of diversification from 10 to 5 Ma that coincides with a period of accelerated elevational niche divergence of Himalayan passerines that reached its peak in the late Pliocene (Price et al., 2014). Along with the establishment of elevational parapatry, the *Carpodacus* rosefinch radiation involved multiple independent “into Tibet” movements, that is, colonizations of the plateau (area E) from its forested fringes (areas F_1_ and F_2_). These were associated with independent colonizations of semiopen and open alpine habitats from ancestral forest habitats (Päckert, Martens, Sun, & Tietze, 2015). In other alpine groups of organisms, there is evidence of the reverse directionality of faunal exchange, that is, from the central plateau to its margins. Indeed, the ancestors of a few Himalayan faunal elements seem to have originated from a Tibetan source area. With increasing aridification and loss of mesophilic forests on the QTP, these Tibetan stem populations dispersed southward into the “Himalayan exile” (area F_1_) where they later diversified as documented for lazy toads (Hoffmann et al., 2017) and ground beetles (Schmidt, Opgenoorth, Höll, & Bastrop, 2012; further examples in Martens, 2015).

### 4.4 Immigration into the QTP region from adjacent source regions

The alpine QTP fauna and flora includes some immigrants from distant regions like the Nearctic (alpine plants: Ebersbach et al., 2017) and the Mediterranean (Clewing, von Oheimb, Vinarski, Wilke, & Albrecht, 2014; Wen et al., 2014), whereas the Himalayan bird communities were apparently shaped by immigration from adjacent subtropical areas in Southeast Asia (Johansson et al., 2007; Päckert et al., 2012).

Among our target groups, some Old World buntings (*Emberiza*) have colonized the eastern plateau margin from a center of origin and diversification northeast of the QTP (area G; similar scenario with higher area uncertainty for bullfinches). Weigold (2005) already supposed that those immigrants from the Northeast were already preadapted to cold and dry environments, given their presumed origin in the arid belt of Central Asian steppes and deserts. Further examples of Eastern Palearctic taxa on the QTP are found in rodents (Li & Wang, 2015). Also, depending on the perspective, the “Out of North China” hypothesis by Zhang et al. (2006) for Asian salamanders (Hynobiidae) can be regarded as an “into Tibet” dispersal. In northern China, successive cyclical vegetation shifts between steppe and forest (or grassland and desert) ecosystems during the Pliocene (Wang et al., 2006), might have furthermore promoted north–south faunal interchange at the eastern QTP margin (Päckert et al., 2012). Only a few of these Eastern Palearctic immigrants actually colonized the Himalayas (two bullfinch species for example; this study), thus these East Siberian/Mongolian taxa are characteristic for the montane and alpine ecosystems of the Hengduanshan (our area F_2_), that is, the eastern QTP margin.

Several authors hypothesized that, as opposed to “centers of origin,” an area can constitute a “center of accumulation,” that is, a region that obtained taxa through unidirectional immigration from different source areas (Goldberg, Roy, Lande, & Jablonski, 2005; Mora, Chittaro, Sale, Kritzer, & Ludsin, 2003). As an equivalent term, an “immigration center” in the Sinohimalayas was suggested by Liu et al. (2017), however, the authors pointed out that evolutionary history of the regional avifauna was shaped by both in situ speciation and immigration. Also, Martens (2015) emphasized that biogeographic affinities of the Himalayas are manifold and during its long uplift history the Himalayan Arc and its extensions in the East did “not merely absorb” immigrant faunal elements from adjacent regions.

## 5 CONCLUSION

Researchers of the early 20th century had already developed clear‐sighted hypotheses on centers of origin and diversification in the QTP region as well as on a Tibetan immigrant mammal fauna from cold and arid environments north of the QTP (Weigold, 1949). This is all the more impressive, because these early biogeographic hypotheses were formulated in times prior to the application of cladistic methods or phylogenetic analysis of molecular data. Our analysis suggests that, in accordance with Weigold's predictions, centers of origin and (in situ) diversification of extant QTP passerine bird genera were not restricted the QTP platform, but also occurred along its southern and southeastern margins (in the Himalayas and the Hengduanshan), and northeast of the QTP in the Eastern Palearctic. Faunal interchange among the QTP, its flanking mountains, and the adjacent regions must therefore have been a bidirectional process rather than a one‐way street (for birds: Cai et al., 2018; Liu et al., 2016; for alpine plants: Ebersbach et al., 2017).

Previous biogeographical studies already acknowledged the fact that faunal interchange between adjacent bioregions in other parts of the world is a “two‐way traffic” (Cheetham, 1963), such as the Great American Biotic Interchange. Although it has been assumed that faunal interchange across the Isthmus of Panama for birds went primarily from south to north (Weir, Bermingham, & Schluter, 2009), recent studies suggested that transcontinental dispersal and colonization of new habitat occurred in both directions (Pelegrin, Gamboa, Menéndez, & Hernández‐Fernández, 2018; Woodburne, 2010). As a result, extant Neotropical faunal assemblages are composed by both native and immigrant taxa (Cione, Gasparini, Soibelzon, Soibelzon, & Tonni, 2015) and this is what Weigold (2005) suggested for the Tibetan avifauna, too. The same pattern of a bidirectional faunal interchange can be expected from future studies that are dedicated either to speciose families with large transcontinental distributions (Ebersbach et al., 2017) or to comparisons of independent radiations across clades of a speciose taxon such as Passeroidea in this study.

## CONFLICT OF INTEREST

No conflict of interest has been declared by the authors.

## AUTHOR CONTRIBUTIONS

**Martin Päckert:** Conceptualization (lead); Data curation (lead); Formal analysis (supporting); Funding acquisition (lead); Investigation (lead); Methodology (supporting); Project administration (lead); Resources (lead); Software (supporting); Supervision (lead); Validation (lead); Visualization (lead); Writing‐original draft (lead); Writing‐review & editing (lead). **Adrien Favre:** Conceptualization (supporting); Formal analysis (equal); Methodology (equal); Software (equal); Visualization (supporting); Writing‐original draft (supporting); Writing‐review & editing (supporting). **Jan Schnitzler:** Formal analysis (supporting); Methodology (supporting); Software (supporting); Validation (supporting); Writing‐review & editing (supporting). **Jochen Martens:** Conceptualization (supporting); Data curation (supporting); Funding acquisition (supporting); Investigation (supporting); Resources (supporting); Supervision (supporting); Writing‐original draft (supporting); Writing‐review & editing (supporting). **Yue‐Hua Sun:** Funding acquisition (supporting); Investigation (supporting); Resources (supporting); Writing‐review & editing (supporting). **Dieter Thomas Tietze:** Conceptualization (supporting); Data curation (supporting); Funding acquisition (supporting); Investigation (supporting); Methodology (supporting); Software (supporting); Validation (supporting); Writing‐review & editing (supporting). **Frank Hailer:** Conceptualization (equal); Funding acquisition (supporting); Investigation (supporting); Methodology (supporting); Writing‐review & editing (supporting). **Ingo Michalak:** Formal analysis (supporting); Methodology (equal); Software (supporting); Validation (supporting); Writing‐review & editing (supporting). **Patrick Strutzenberger:** Conceptualization (supporting); Data curation (supporting); Formal analysis (equal); Investigation (supporting); Methodology (equal); Software (equal); Supervision (supporting); Validation (supporting); Writing‐original draft (supporting); Writing‐review & editing (supporting).

## Supporting information

Fig S1

Fig S2

Fig S3

Fig S4

Supplementary Material

## Data Availability

All newly generated sequences have been submitted to GenBank (accession numbers: see Table S3). The tree file and further information such as data matrices (e.g., area matrices) have been deposited on Dryad under https://doi.org/10.5061/dryad.xksn02vd0.
