# Supplementary material for: “Into and Out of” the Qinghai‐Tibet Plateau and the Himalayas: Centers of origin and diversification across five clades of Eurasian montane and alpine passerine birds
Source: Ecol Evol. 2020 Aug 4;10(17):9283–300. doi: 10.1002/ece3.6615 (PMC7487248; doi:10.1002/ece3.6615)

Into and Out of' the Qinghai Tibet Plateau and the Himalayas: centers of origin and diversification compared across five clades of Eurasian montane and alpine passerine birds

Martin Päckert, Adrien Favre, Jan Schnitzler, Jochen Martens, Yue-Hua Sun
Dieter Thomas Tietze, Frank Hailer, Ingo Michalak, Patrick Strutzenberger

Figure S3

Ancestral range reconstruction fitting the DEC model to a time-calibrated tree of Passeroidea (275 ingroup species). The time-calibrated phylogenetic tree was generated in BEAST, using eight calibration points (fossils). The biogeographic analysis included a total of eight areas (A-H, see map); best states shown for ancestral ranges; outgroup *Bombycilla*: Holarctic distribution of the genus areas A= *B. cedrorum*; BG= *B. garrulus* + *B. japonica*)

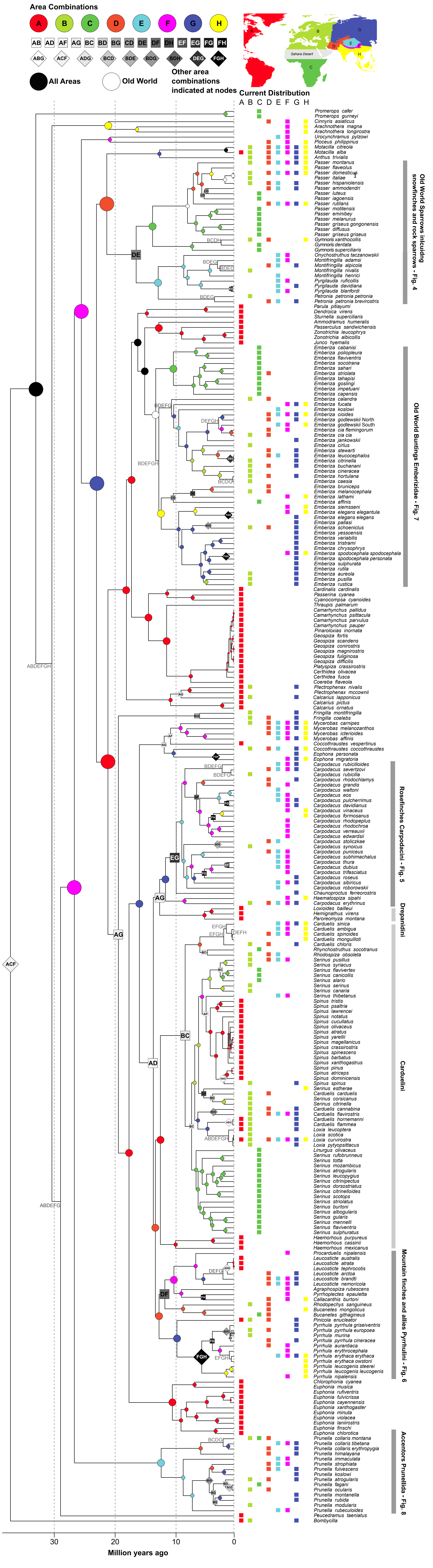

Supplement: Supplementary file 3 — Fig S3 [file ECE3-10-9283-s003.pdf]
