# Supplementary material for: “Into and Out of” the Qinghai‐Tibet Plateau and the Himalayas: Centers of origin and diversification across five clades of Eurasian montane and alpine passerine birds
Source: Ecol Evol. 2020 Aug 4;10(17):9283–300. doi: 10.1002/ece3.6615 (PMC7487248; doi:10.1002/ece3.6615)

‘Into and Out of’ the Qinghai-Tibet Plateau and the Himalayas: centers of origin and diversification compared across five clades of Eurasian montane and alpine passerine birds

Martin Päckert, Adrien Favre, Jan Schnitzler, Jochen Martens, Yue-Hua Sun
Dieter Thomas Tietze, Frank Hailer, Ingo Michalak, Patrick Strutzenberger

Figure S4

Ancestral range reconstruction fitting the DEC model to a time-calibrated tree of Passeroidea (275 ingroup species).
The time-calibrated phylogenetic tree was generated in BEAST, using eight calibration points (fossils).
The biogeographic analysis included a total of eight areas; per-area probabilities shown for ancestral ranges;
outgroup *Bombycilla*: Holarctic distribution of the genus areas A= *B. cedrorum*; BG= *B. garrulus* + *B. japonica*)

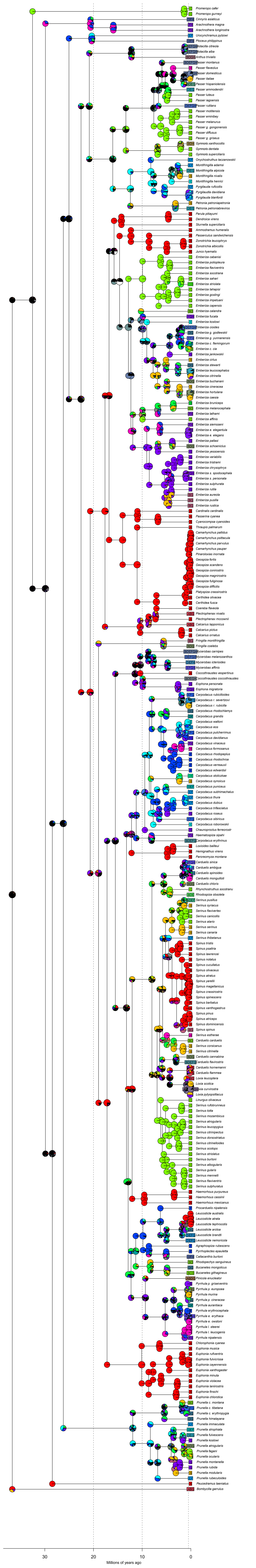

Supplement: Supplementary file 4 — Fig S4 [file ECE3-10-9283-s004.pdf]
