# Supplementary material for: “Into and Out of” the Qinghai‐Tibet Plateau and the Himalayas: Centers of origin and diversification across five clades of Eurasian montane and alpine passerine birds
Source: Ecol Evol. 2020 Aug 4;10(17):9283–300. doi: 10.1002/ece3.6615 (PMC7487248; doi:10.1002/ece3.6615)
Supplement: Supplementary file 5 — Supplementary Material [file ECE3-10-9283-s005.docx]

**‘Into and Out of’ the Qinghai-Tibet Plateau and the Himalayas: centers of origin and diversification compared across five clades of Eurasian montane and alpine passerine birds**

Martin Päckert, Adrien Favre, Jan Schnitzler, Jochen Martens, Yue-Hua Sun, Dieter Thomas Tietze, Frank Hailer, Ingo Michalak, Patrick Strutzenberger

**Appendix 1: Supplementary Tables**

**Table S1** Into-Tibet (Himalayas) and Out-of-Tibet (Himalayas) scenarios postulated for different terrestrial organisms; methods of ancestral-range reconstruction (ARR) indicated, if performed in the according study (including the number of areas); inference of areas of origin and diversification in other studies (ARR = no) relied either on time horizons and spatial distribution of the fossil record or on mere deductive inference of ancestral areas from the tree topology and distribution of tip taxa (‘phylogenetic’).

| **Out of (Sino-)Himalayas** | | | | | | | | |
| --- | --- | --- | --- | --- | --- | --- | --- | --- |
| **group** | **study taxon** | **source area [area in this study]** | **sink area [area in this study]** | **ARR** | **method** | **n areas** | **range** | **reference** |
| birds | accentors, *Prunella* | Himalayas [F_1_] | Palearctic [B, D, G] | yes | Lagrange | 4 | regional | Drovetski et al*.,* 2013 |
| birds | accentors, *Prunella* | Sinohimalayas [F], Central Asia [D] and parts of Mongolia [D] | Palearctic [B, D, G] | yes | RASP | 6 | regional | Liu et al*.,* 2017 |
| birds | redstarts, *Phoenicurus* | Sinohimalayas [F] | Palearctic, Oriental [B, D, G, H] | yes | Lagrange | 6 | regional | Voelker et al*.*, 2015 |
| birds | tits, Paridae | China [F_2_, parts of H] | Holarctic, Afrotropics, Oriental [nearly all] | yes | S-DIVA, Lagrange | 10 | global | Tietze & Borthakur, 2012 |
| birds | tits, Paridae | Sinohimalayas [F, parts of H] | Holarctic, Afrotropics, Oriental [nearly all] | yes | BioGeoBEARS | 10/11 | global | Johansson et al*.,* 2018 |
| birds | babblers | Sinohimalayas [F] | Old World including Afrotropics and Australia | yes | BioGeoBEARS |  | global | Cai et al. 2020 |
| mammals | Rodentia, Dipodoidea | Himalayas, Tibet, Central Asia  [D, E, F_1_, H] | Palearctic [B, D, G] | yes | Lagrange | 9 | global | Pisano et al*.,* 2016 |
| plants | *Myricaria* | Sinohimalayas (+Pamir-Alay) [D, F] | Palearctic [B, G, A_1_] | yes | Lagrange | 6 | regional | Zhang et al*.,* 2014 |
| **Out of Tibet** | | | | | | | | |
| **group** | **study taxon** | **source area [area in this study]** | **sink area [area in this study]** | **ARR** | **method** | **n area** | **range** | **Reference** |
| amphibia | lazy toads, *Scutiger* | paleo-Tibet [E] | Sinohimalayas [F] | no | phylogenetic | - | - | Hoffmann et al*.,* 2017 |
| beetles | *Ethira* | Tibet [E] | Sinohimalayas [F] | no | phylogenetic | - | - | Schmidt et al*.,* 2012 |
| mammals | foxes | Tibet [E] | Palearctic [B, D, G] | no | fossil record | - | - | Wang et al. 2014 |
| mammals | wooly rhinos | Tibet [E] | Palearctic [B, D, G] | no | fossil record | - | - | Deng et al. 2011 |
| mammals | sheep, *Protovis* | Tibet [E] | Palearctic [B, D, G] | no | fossil record | - | - | Wang et al. 2016 |
| mammals | Hyenas, *Chasmaporthetes* | Tibet [E] | Palearctic [B, D, G] | no | fossil record | - | - | Tseng et al. 2014 |
| plants | *Gentiana* | Tibet and Central Asian Mts [D, E] | Holarctic, Australasian  [nearly all] | yes | BioGeoBEARS | 11 | global | Favre et al*.*, 2016 |
| **Into Tibet/into Himalayas** | | | | | | | | |
| **group** | **study taxon** | **source area [area in this study]** | **sink area [area in this study]** | **ARR** | **method** | **n area** | **range** | **reference** |
| molluscs | Valvata | Euro-Mediterranean [B] | QTP and margins [E, F] | yes | Lagrange | 5 | Holarctic | Clewing et al*.,* 2011 |
| plants | *Diapensia* | Arctic, Japan [G] | Himalayan-Hengduan Mountains [F] | yes | S-DIVA | 3 | regional | Hou et al*.,* 2016 |
| mammals | Rodentia, Spalacidae | North China, Mongolian Plateau [G] | Tibet [E] | no | fossil record | - | - | Li & Wang, 2015 |
| amphibians | Hynobiidae | North China [G] | eastern QTP margin [E, F_2_] and other adjacent regions [H] | no | phylogenetic | - | - | Zhang et al*.,* 2006 |
| **Bidirectional** | | | | | | | | |
| **group** | **study taxon** | **source area** | **sink area [area in this study]** | **ARR** | **method** | **n area** | **range** | **reference** |
| plants | *Saxifraga* | several independent | QTP and margins [E, F] | yes | BioGeoBEARS | 10 | global | Ebersbach et al*.,* 2017 |
| Aves | parrotbills, *Paradoxornis* | Sinohimalayan, Indo-Burma [F, H] | East Asia [G] | yes | BioGeoBEARS | 4 | regional | Liu et al*.,* 2016 |

**Table S2** Samples and sequences used for phylogenetic reconstruction; taxon names according to two taxonomic standards (Clements et al., 2015 and Gill & Donsker, 2018); sequences newly generated for this study marked in bold (accession nos to be provided upon acceptance); collections who donated samples for this study: MTD = Senckenberg Natural History Collections Dresden, Museum of Zoology, Germany; ZMB = Museum für Naturkunde Berlin; ZFMK = Zoologisches Forschungsmuseum Alexander Koenig Bonn, Germany; NME = Naturkundemuseum Erfurt, Germany; BMNH = Natural History Museum at Tring, Bird Group, UK; ZMUC = Zoological Museum of the University of Copenhagen, Denmark (NHMD = Natural History Museum of Denmark); NHMC = Natural History Museum of Crete, Greece; UWBM = Burke Museum of Natural History and Culture, Seattle, USA – further collection acronyms (GenBank sequences): NRM = Natural History Museum of Stockholn, Sweden; AJN = Ajtte Swedish Mountain and Sami Museum, Jokkmokk, Sweden; ZMA = Zoological Museum of Amsterdam, The Netherlands; RMNH = Naturalis Biodiversity Center, Leiden, The Netherlands; MRSNT = Museo di Storia Naturale, Torino, Italy; AMNH = American Museum of Natural History, NY, USA; USNM = National Museum of Natural History, Smithsonian Institution, Washington D.C., USA; FMNH = The Field Museum of Natural History, Chicago, USA; LSUMZ = Lousiana Museum of Natural History, Baton Rouge, USA; MVZ = Museum of Vertebrate Zoology, University of California, Berkley, USA; BMNH# = Bell Museum of Natural History, Minneapolis, USA; KU = Kansas University, USA; CAS/IOZ = Chinese Academy of Sciences, Institute of Zoology, Beijing, China; NMNS = National Museum of Natural Science, Taipei, Taiwan.

| **sample no** | **Clements et al. (2015)** | **Gill & Donsker (2018)** | **family** | **country** | **voucher** | **cytb** | **ND2** | **myo2** | **ODC** |
| --- | --- | --- | --- | --- | --- | --- | --- | --- | --- |
| GenBank | *Acanthisitta chloris* | *Acanthisitta chloris* | Acanthisittidae | Australia | NRM569989 | AY325307 | AY325307 | EU726212 | EU726220 |
| GenBank | *Bombycilla garrulus* | *Bombycilla garrulus* | Bombycillidae | Sweden | NRM 986044 | AY228049 | DQ466855 | AY228286 | EU680709 |
| GenBank | *Cardinalis cardinalis* | *Cardinalis cardinalis* | Cardinalidae | USA | NRM 20036311 | EU325777 | - | KC313784 | EU325835 |
| GenBank | *Cyanocompsa cyanoides* | *Cyanocompsa cyanoides* | Cardinalidae | Bolivia | LSUMNS B-12708 | AF301462 | EF529889* | EU191805 | EU191813 |
| GenBank | *Passerina cyanea* | *Passerina cyanea* | Cardinalidae | USA | LSUMZ B-20773 | AF290148 | AF290111 | EU191799 | EU191807 |
| 100178 | *Emberiza affinis* | *Emberiza affinis* | Emberizidae | Nigeria | BMNH 1976.2.1 | KP877712 | - | - | - |
| MAR5927 | *Emberiza aureola* | *Emberiza aureola* | Emberizidae | Russia |  | KP877778 | - | KP877870 | **MT336192** |
| MAR6625 | *Emberiza bruniceps* | *Emberiza bruniceps* | Emberizidae | Iran | NHMD 137739 | KP877782 | KX109685 | KP877874 | KX109725 |
| GenBank | *Emberiza buchanani* | *Emberiza buchanani* | Emberizidae | Kazakhstan |  | EU325757 | - | - | EU325815 |
| GenBank | *Emberiza cabanisi* | *Emberiza cabanisi* | Emberizidae | Cameroon |  | EU325767 | - | JX515372 | EU325825 |
| MAR6627 | *Emberiza caesia* | *Emberiza caesia* | Emberizidae | Turkey |  | KP877784 | MT210138 | KP877875 | **MT336193** |
| MAR696 | *Emberiza calandra* | *Emberiza calandra* | Emberizidae | Germany |  | KP877792 | KX109686 | KP877878 | KX109726 |
| GenBank | *Emberiza capensis* | *Emberiza capensis* | Emberizidae | South Africa |  | EU325765 | - | JX515375 | EU325823 |
| MAR1473 | *Emberiza chrysophrys* | *Emberiza chrysophrys* | Emberizidae | Russia |  | KP877719 | MT210146 | KP877844 | EU325791* |
| MAR2586 | *Emberiza cia cia* | *Emberiza cia cia* | Emberizidae | Germany | MTD C61448 | KP877723 | MT210137 | KP877848 | **MT336183** |
| MAR3346 | *Emberiza cia flemingorum* | *Emberiza cia flemingorum* | Emberizidae | Nepal | NME 04/035 | KP877725 | - | KP877849 | - |
| NHMC 80.4.193.11 | *Emberiza cineracea* | *Emberiza cineracea* | Emberizidae | Greece | NHMC 80.4.193.11 | KP877806 | MT210121 | KP877882 | **MT336195** |
| MAR655 | *Emberiza cioides* | *Emberiza cioides* | Emberizidae | aviary | MTD C55352 | KP877780 | MT210120 | KP877872 | **MT336177** |
| MAR5569 | *Emberiza cirlus* | *Emberiza cirlus* | Emberizidae | France |  | KP877767 | - | KP877866 | **MT336189** |
| MAR3479 | *Emberiza citrinella* | *Emberiza citrinella* | Emberizidae | Germany |  | KP877727 | KX109687 | KP877851 | KX109727 |
| MAR4475 | *Emberiza elegans elegans* | *Emberiza elegans elegans* | Emberizidae | Russia |  | KP877742 | KX109688 | KP877856 | KX109728 |
| MAR702 | *Emberiza elegans elegantula* | *Emberiza elegans elegantula* | Emberizidae | China | MTD C56734 | KP877797 | MT210122 | KP877880 | **MT336179** |
| MAR662 | *Emberiza flaviventris* | *Emberiza flaviventris* | Emberizidae | aviary | MTD C53594 | KP877781 | KX109689 | KP877873 | KX109729 |
| MAR1246 | *Emberiza fucata* | *Emberiza fucata* | Emberizidae | Russia |  | KP877716 | - | KP877843 | **MT336180** |
| MAR5770 | *Emberiza godlewskii godlewskii* | *Emberiza godlewskii godlewskii* | Emberizidae | China |  | KP877770 | MT210123 | KP877868 | **MT336190** |
| MAR4952 | *Emberiza godlewskii yunnanensis* | *Emberiza godlewskii yunnanensis* | Emberizidae | China | MTD C62973 | KP877762 | MT210124 | KP877865 | **MT336188** |
| GenBank | *Emberiza goslingi* | *Emberiza goslingi* | Emberizidae | Nigeria |  | JX515341 | - | JX515378 | JX515356 |
| MAR557 | *Emberiza hortulana* | *Emberiza hortulana* | Emberizidae | Germany |  | KP877768 | - | KP877867 | **MT336176** |
| MAR4541 | *Emberiza impetuani* | *Emberiza impetuani* | Emberizidae | Namibia |  | KP877747 | KX109690 | KP877857 | KX109730 |
| GenBank | *Emberiza jankowskii* | *Emberiza jankowskii* | Emberizidae | China |  | EU325761 | - | - | EU325819 |
| MAR9356 | *Emberiza koslowi* | *Emberiza koslowi* | Emberizidae | China |  | **MT210115** | - | KP877881 | **MT336194** |
| GenBank | *Melophus lathami* | *Emberiza lathami* | Emberizidae | Nepal | AMNH Birds DOT-5536 | KJ456338 | - | KJ454831 | EU325808* |
| MAR1627 | *Emberiza leucocephalos* | *Emberiza leucocephalos* | Emberizidae | Kyrgyzstan | MTD C63035 | KP877721 | MT210139 | KP877846 | **MT336182** |
| MAR6632 | *Emberiza melanocephala* | *Emberiza melanocephala* | Emberizidae | Greece | NHMD 132240 | KP877787 | KX109691 | KP877876 | KX109731 |
| MAR4884 | *Emberiza pallasi polaris* | *Emberiza pallasi polaris* | Emberizidae | Russia |  | KP877757 | MT210125 | KP877862 | **MT336186** |
| 100163 | *Emberiza poliopleura* | *Emberiza poliopleura* | Emberizidae | Ethiopia | ZMB 2000.11463 | KP877710 | - | - | - |
| MAR4898 | *Emberiza pusilla* | *Emberiza pusilla* | Emberizidae | Russia |  | KP877759 | MT210126 | KP877863 | **MT336187** |
| MAR3968 | *Emberiza rustica* | *Emberiza rustica* | Emberizidae | Finland |  | KP877729 | KX109692 | KP877853 | KX109732 |
| MAR4867 | *Emberiza rutila* | *Emberiza rutila* | Emberizidae | Russia |  | KP877754 | - | KP877860 | **MT336184** |
| MAR170 | *Emberiza sahari* | *Emberiza sahari* | Emberizidae | Morocco |  | KP877722 | - | KP877847 | JX515359* |
| MAR612 | *Emberiza schoeniclus* | *Emberiza schoeniclus* | Emberizidae | Russia |  | KP877779 | KX109693 | KP877871 | KX109733 |
| MAR4901 | *Latoucheornis siemsseni* | *Emberiza siemsseni* | Emberizidae | China |  | KP877760 | - | KP877864 | KX109734 |
| 100179 | *Emberiza socotrana* | *Emberiza socotrana* | Emberizidae | Yemen | BMNH 1953.36.35 | KP877713, KJ5118004) | - | KJ511801 | **-** |
| MAR4352 | *Emberiza spodocephala personata* | *Emberiza spodocephala personata* | Emberizidae | Russia |  | KP877738 | MT210127 | KP877855 | - |
| MAR337 | *Emberiza spodocephala spodocephala* | *Emberiza spodocephala spodocephala* | Emberizidae | Russia |  | KP877726 | KX109694 | KP877850 | KX109735 |
| MAR1597 | *Emberiza stewarti* | *Emberiza stewarti* | Emberizidae | Kyrgyzstan | MTD C63036 | KP877720 | - | KP877845 | **MT336181** |
| GenBank | *Emberiza striolata* | *Emberiza striolata* | Emberizidae | Israel | NRM 20076357 | EU325762 | - | JX515386 | EU325820 |
| GenBank | *Emberiza sulphurata* | *Emberiza sulphurata* | Emberizidae |  |  | EU325737 | - | - | EU325795 |
| MAR700 | *Emberiza tahapisi* | *Emberiza tahapisi* | Emberizidae | PR Yemen |  | KP877796 | - | JX515389 | **MT336178** |
| MAR5912 | *Emberiza tristrami* | *Emberiza tristrami* | Emberizidae | Russia |  | KP877775 | MT210131 | KP877869 | **MT336191** |
| GenBank | *Emberiza variabilis* | *Emberiza variabilis* | Emberizidae | Japan |  | EU325734 | - | - | EU325792 |
| MAR4883 | *Emberiza yessoensis* | *Emberiza yessoensis* | Emberizidae | Russia |  | KP877756 | MT210128 | KP877861 | **MT336185** |
| GenBank | *Amandava amandava* | *Amandava amandava* | Estrildidae |  | NHMD135615 | KJ456191 | KJ455319 | KJ454750 | KJ455720 |
| GenBank | *Acanthis flammea* | *Acanthis flammea* | Fringillidae | Sweden | NRM 20016449 | L76386 | JN715417 | JN715235 | JN715325 |
| GenBank | *Acanthis hornemanni* | *Acanthis hornemanni* | Fringillidae | Sweden | AJN 000043 | U83201 | JN715419 | JN715237 | JN715327 |
| MAR90161 | *Carpodacus rubescens* | *Agraphospiza rubescens* | Fringillidae | Nepal |  | KF194120 | KF194163 | KF194025 | KX109721 |
| MAR1172 | *Bucanetes githagineus* | *Bucanetes githagineus* | Fringillidae | Spain | MTD C64524 | HQ284701 | KX109683 | KX109664 | KX109716 |
| UWBM46256 | *Bucanetes mongolicus* | *Bucanetes mongolicus* | Fringillidae | Mongolia | UWBM46256 | **MT210116** | MT210129 | JN715295 | JN715387 |
| GenBank | *Callacanthis burtoni* | *Callacanthis burtoni* | Fringillidae | India | NRM 570789 | - | JN715409 | JN715227 | JN715317 |
| GenBank | *Carduelis carduelis* | *Carduelis carduelis* | Fringillidae | Sweden | NRM 996076 | EU325788 | JN715414 | JN715232 | JN715322 |
| GenBank | *Serinus citrinella* | *Carduelis citrinella* | Fringillidae | Liechtenstein | NRM 553307 | L77872 | JN715481 | JN715299 | JN715391 |
| GenBank | *Serinus corsicanus* | *Carduelis corsicana* | Fringillidae | Italy |  | AY583725 | - | - | - |
| MAR6907 | *Carpodacus davidianus* | *Carpodacus davidianus* | Fringillidae | China | MTD C64235 | KF194060 | KF194156 | KF194018 | **MT336196** |
| MAR8141 | *Carpodacus dubius* | *Carpodacus dubius* | Fringillidae | China |  | KF194111 | KF194144 | KF194023 | **-** |
| FM8 | *Carpodacus edwardsii* | *Carpodacus edwardsii* | Fringillidae | Nepal | FMNH277070 | KF194170 | - | - | KJ455732 |
| MAR8157 | *Carpodacus eos* | *Carpodacus waltoni eos* | Fringillidae | China |  | KF194068 | KF194143 | KF194032 | **MT336197** |
| MAR1635 | *Carpodacus erythrinus* | *Carpodacus erythrinus* | Fringillidae | Kyrgyzstan | MTD C63043 | KF194047 | KF194157 | KF194028 | KX109718 |
| GenBank | *Chaunoproctus ferreorostris* | *Carpodacus ferreorostris* | Fringillidae | Bonin | BMNH 1855.12.19.71 | - | JN715445 | - | - |
| T1275 | *Carpodacus formosanus* | *Carpodacus formosanus* | Fringillidae | Taiwan |  | KF194119 | KF194167 | KF194006 | **MT336198** |
| MAR5349 | *Carpodacus grandis* | *Carpodacus grandis* | Fringillidae | Nepal | ZFMK J.II.22.g1.g | KF194051 | KF194152 | KF194010 | - |
| MAR3639 | *Carpodacus pulcherrimus* | *Carpodacus pulcherrimus* | Fringillidae | China | MTD C64234 | KF194054 | KF194154 | KF194015 | KX109720 |
| FM33 | *Carpodacus puniceus* | *Carpodacus puniceus* | Fringillidae | Nepal | FMNH 277071 | KF194173 | KF194132 | JN715250 | JN715340 |
| NRM21 | *Carpodacus rhodochlamys* | *Carpodacus rhodochlamys* | Fringillidae |  | NRM 20026491 | KF194174 | KF194133 | KF194035 | KJ455734 |
| MAR3331 | *Carpodacus rodochroa* | *Carpodacus rodochroa* | Fringillidae | Nepal | NME 03/71 | KF194080 | KF194161 | KF194014 | JN715344 |
| AM33 | *Carpodacus rodopeplus* | *Carpodacus rodopeplus* | Fringillidae | Nepal | AMNH DOT 5631 | KF194177 | KF194125 | KF194033 | JN715345 |
| MAR2322 | *Carpodacus roseus* | *Carpodacus roseus* | Fringillidae | captivity | MTD C61316 | KF194085 | KF194162 | KF194024 | **MT336199** |
| MAR5459 | *Carpodacus rubicilla* | *Carpodacus rubicilla* | Fringillidae | Russia | ZFMK J.II.22.f.l | KF194088 | KF194148 | KF194011 | - |
| UWBM76129 | *Carpodacus rubicilla severtzovi* | *Carpodacus rubicilla severtzovi* | Fringillidae | Russia | UWBM76129 | **MT210118** | MT210133 | MT277429 | MT336200 |
| MAR5931 | *Carpodacus rubicilloides* | *Carpodacus rubicilloides* | Fringillidae | China |  | KF194089 | KF194145 | KF194016 | JN715349 |
| MAR1240 | *Uragus sibiricus* | *Carpodacus sibiricus* | Fringillidae | Russia |  | KF194095 | KF194168 | KF194013 | JN715404 |
| GenBank | *Haematospiza sipahi* | *Carpodacus sipahi* | Fringillidae | India | NRM 570790 | AF342875 | JN715456 | JN715274 | - |
| BMNH1967.17803 | *Carpodacus stoliczkae* | *Carpodacus stoliczkae* | Fringillidae |  | BMNH1967.17803 | KF194103 | KF194147 | KF194009 | - |
| MAR5088 | *Carpodacus synoicus* | *Carpodacus synoicus* | Fringillidae | Jordan | - | KF194102 | KF194149 | KF194031 | KX109722 |
| AM21 | *Carpodacus thura* | *Carpodacus thura* | Fringillidae | Nepal | AMNH DOT 5609 | KF194181 | KF194135 | KF194034 | KJ455736 |
| MAR6939 | *Carpodacus trifasciatus* | *Carpodacus trifasciatus* | Fringillidae | China | MTD C63834 | KF194115 | KF194166 | KF194019 | **MT336201** |
| CA8 | *Carpodacus verreauxii* | *Carpodacus verreauxii* | Fringillidae | China | CAS ORN 95886 | KF194169 | KF194128 | KF194033 | - |
| MAR8115 | *Carpodacus vinaceus* | *Carpodacus vinaceus* | Fringillidae | China | MTD C64244 | KF194118 | KF194142 | KF194022 | KX109723 |
| MAR5936 | *Carpodacus waltoni* | *Carpodacus waltoni* | Fringillidae | China |  | KF194057 | KF194141 | KF194017 | - |
| GenBank | *Chloris ambigua* | *Chloris ambigua* | Fringillidae | captivity | NRM20026539 | U78322 | JN715410 | JN715228 | JN715318 |
| GenBank | *Chloris chloris* | *Chloris chloris* | Fringillidae | Sweden | NRM 986328 | L76297 | JN715415 | JN715233 | JN715323 |
| GenBank | *Chloris monguilloti* | *Chloris monguilloti* | Fringillidae | Vietnam | NRM 546196 | - | JN715421 | JN715239 | JN715329 |
| GenBank | *Chloris sinica* | *Chloris sinica* | Fringillidae | captivity | NRM 20026538 | L76592 | JN715424 | JN715242 | JN715332 |
| GenBank | *Chloris spinoides* | *Chloris spinoides* | Fringillidae | captivity | NRM 20026503 | U79018 | JN715425 | JN715243 | JN715333 |
| GenBank | *Chlorophonia cyanea* | *Chlorophonia cyanea* | Fringillidae | captivity | NRM 20066989 | - | - | JN715263 | JN715353 |
| GenBank | *Serinus estherae* | *Chrysocorythus estherae* | Fringillidae | Java | RMNH 44712 | - | JN715483 | - | - |
| MTD127 | *Coccothraustes coccothraustes* | *Coccothraustes coccothraustes* | Fringillidae | Germany | MTD C61158 | HQ284693 | KX109684 | KX109665 | KX109724 |
| GenBank | *Coccothraustes vespertinus* | *Coccothraustes vespertinus* | Fringillidae | USA | NRM 570795 | EF530032 | JN715457 | JN715275 | JN715367 |
| GenBank | *Serinus albogularis* | *Crithagra albogularis* | Fringillidae | South Africa |  | L78705 | - | - | - |
| GenBank | *Serinus atrogularis* | *Crithagra atrogularis* | Fringillidae | South Africa |  | L76267 | - | - | - |
| GenBank | *Serinus burtoni* | *Crithagra burtoni* | Fringillidae | Nigeria | NRM 20086267 | AY790896 | JN715479 | JN715297 | JN715389 |
| GenBank | *Serinus citrinelloides* | *Crithagra citrinelloides* | Fringillidae | Captivity | NRM 20026501 | L77555 | JN715482 | JN715300 | JN715392 |
| GenBank | *Serinus citrinipectus* | *Crithagra citrinipectus* | Fringillidae | Mozambique |  | L78707 | - | - | - |
| GenBank | *Serinus dorsostriatus* | *Crithagra dorsostriata* | Fringillidae | Tanzania |  | L76278 | - | - | - |
| GenBank | *Serinus flaviventris* | *Crithagra flaviventris* | Fringillidae | South Africa |  | L76280 | - | - | - |
| GenBank | *Serinus gularis* | *Crithagra gularis* | Fringillidae | South Africa |  | L77556 | - | - | - |
| GenBank | *Serinus leucopygius* | *Crithagra leucopygia* | Fringillidae | Nigeria | NRM 20106050 | L76264 | JN715485 | JN715302 | JN715394 |
| GenBank | *Serinus mennelli* | *Crithagra mennelli* | Fringillidae | Captivity | NRM 20026500 | - | JN715486 | JN715303 | JN715395 |
| GenBank | *Serinus mozambicus* | *Crithagra mozambica* | Fringillidae | Swaziland | NRM 20066026 | L76265 | JN715487 | JN715304 | JN715396 |
| GenBank | *Serinus rufobrunneus* | *Crithagra rufobrunnea* | Fringillidae | Bioko | NRM 857618 | - | JN715489 | JN715306 | JN715398 |
| GenBank | *Serinus scotops* | *Crithagra scotops* | Fringillidae |  |  | AY790894 | - | - | - |
| GenBank | *Serinus striolatus* | *Crithagra striolata* | Fringillidae | DR Congo | NRM 570782 | L77557 | JN715491 | JN715308 | JN715400 |
| GenBank | *Serinus sulphuratus* | *Crithagra sulphurata* | Fringillidae | Captivity | NRM 20026498 | L76294 | JN715492 | JN715309 | JN715401 |
| GenBank | *Pseudochloroptila totta* | *Crithagra totta* | Fringillidae | South Africa |  | AY570548 | - | - | - |
| GenBank | *Eophona migratoria* | *Eophona migratoria* | Fringillidae | Russia | NRM 896473 | AY495388 | JN715447 | JN715264 | JN715355 |
| GenBank | *Eophona personata* | *Eophona personata* | Fringillidae |  | KU 4239 | MF458374 | MF458394 | - | MF458430 |
| GenBank | *Euphonia cayennensis* | *Euphonia cayennensis* | Fringillidae | captivity | NRM 20056062 | - | - | JN715265 | JN715356 |
| GenBank | *Euphonia chlorotica* | *Euphonia chlorotica* | Fringillidae | Paraguay | NRM 956750 | AY228060 | JN715448 | AY228298 | JN715357 |
| GenBank | *Euphonia finschi* | *Euphonia finschi* | Fringillidae | captivity | NRM 20066306 | AF290143 | - | JN715270 | JN715362 |
| GenBank | *Euphonia fulvicrissa* | *Euphonia fulvicrissa* | Fringillidae |  |  | AF383014 | AF383130 | - | - |
| GenBank | *Euphonia laniirostris* | *Euphonia laniirostris* | Fringillidae | captivity | NRM 20066309 | AF006232 | JN715449 | JN715266 | JN715358 |
| GenBank | *Euphonia minuta* | *Euphonia minuta* | Fringillidae | captivity | NRM 20066307 | - | JN715450 | JN715267 | JN715359 |
| GenBank | *Euphonia musica* | *Euphonia musica* | Fringillidae | Paraguay | NRM 976696 | AF310067 | JN715451 | JN715268 | JN715360 |
| GenBank | *Euphonia rufiventris* | *Euphonia rufiventris* | Fringillidae | captivity | NRM 20066310 | - | JN715452 | JN715269 | JN715361 |
| GenBank | *Euphonia violacea* | *Euphonia violacea* | Fringillidae | Paraguay | NRM 966943 | - | JN715453 | JN715271 | JN715363 |
| GenBank | *Euphonia xanthogaster* | *Euphonia xanthogaster* | Fringillidae | captivity | NRM 20066305 | - | JN715454 | JN715272 | JN715364 |
| MTD101 | *Fringilla coelebs* | *Fringilla coelebs* | Fringillidae | Germany | MTD C61071 | HQ284698 | KX109695 | KF194007 | KX109736 |
| GenBank | *Fringilla montifringilla* | *Fringilla montifringilla* | Fringillidae | Sweden | NRM 20046395 | AY495390 | GU816851 | GU816941 | GU816920 |
| MAR92008 | *Haemorhous cassini* | *Haemorhous cassini* | Fringillidae | USA | MVZ169316 | KF194124 | KF194151 | KF194029 | - |
| MAR92009 | *Haemorhous mexicanus* | *Haemorhous mexicanus* | Fringillidae | USA | MVZ 170386 | KF194123 | KF194158 | KF194026 | KX109737 |
| MAR92012 | *Haemorhous purpureus* | *Haemorhous purpureus* | Fringillidae | USA | MVZ 171671 | KF194122 | KF194160 | KF194027 | KX109738 |
| GenBank | *Hemignathus virens* | *Hemignathus virens* | Fringillidae | USA | RCF 2913 | AF015755 | JN715496 | JN715313 | JN715405 |
| UWBM57878 | *Leucosticte arctoa* | *Leucosticte arctoa* | Fringillidae | USA | UWBM57878 | **MT210117** | MT210130 | **MT277430** | **MT336202** |
| GenBank | *Leucosticte atrata* | *Leucosticte atrata* | Fringillidae | USA |  | - | FJ547680 | - | - |
| GenBank | *Leucosticte australis* | *Leucosticte australis* | Fringillidae | USA |  | - | FJ547631 | - | - |
| MAR3634 | *Leucosticte brandti* | *Leucosticte brandti* | Fringillidae | China |  | HQ284699 | KX109696 | KX109666 | KX109739 |
| MAR4246 | *Leucosticte nemoricola* | *Leucosticte nemoricola* | Fringillidae | Nepal | MTD C62013 | HQ284700 | KF194139 | KF194008 | KX109740 |
| SVD 2371 | *Leucosticte tephrocotis* | *Leucosticte tephrocotis* | Fringillidae | USA | UWBM66944 | KX109627 | KX109697 | KX109667 | KX109741 |
| GenBank | *Carduelis cannabina* | *Linaria cannabina* | Fringillidae | Sweden | NRM 966403 | L76298 | JN715413 | JN715231 | JN715321 |
| GenBank | *Carduelis flavirostris* | *Linaria flavirostris* | Fringillidae | Sweden | NRM 20066634 | U83199 | JN715418 | JN715236 | JN715326 |
| GenBank | *Lingurus olivaceus* | *Lingurus olivaceus* | Fringillidae | Nigeria | NRM 20086232 | - | JN715462 | JN715280 | JN715372 |
| GenBank | *Loxia curvirostra* | *Loxia curvirostra* | Fringillidae | Sweden | NRM 976546 | AY228065 | GU816852 | AY228303 | GU816921 |
| GenBank | *Loxia leucoptera* | *Loxia leucoptera* | Fringillidae | Sweden | NRM 20026565 | AF342878 | JN715463 | JN715281 | JN715373 |
| GenBank | *Loxia pytyopsittacus* | *Loxia pytyopsittacus* | Fringillidae | Sweden | NRM 20046001 | AF171664 | JN715464 | JN715282 | JN715374 |
| GenBank | *Loxia scotica* | *Loxia scotica* | Fringillidae | UK |  | AF171656 | - | - | - |
| GenBank | *Loxioides bailleui* | *Loxioides bailleui* | Fringillidae | USA | MRSNT 5783 | EU600383 | JN715465 | JN715283 | JN715375 |
| GenBank | *Mycerobas affinis* | *Mycerobas affinis* | Fringillidae |  | AMNH 5592 | KJ456354 | KJ455517 | - | - |
| GenBank | *Mycerobas carnipes* | *Mycerobas carnipes* | Fringillidae | China | NRM 570797 | AF342880 | JN715466 | JN715284 | JN715376 |
| GenBank | *Mycerobas icterioides* | *Mycerobas icterioides* | Fringillidae |  | FMNH:256357 | KJ456356 | - | - | - |
| GenBank | *Mycerobas melanozanthus* | *Mycerobas melanozanthos* | Fringillidae | Nepal | FMNH 222122 | KJ456357 | KJ455519 | - | - |
| GenBank | *Paroreomyza montana* | *Paroreomyza montana* | Fringillidae | USA | RCF 1984 | AF015759 | JN715467 | JN715285 | JN715377 |
| Pine3 | *Pinicola enucleator* | *Pinicola enucleator* | Fringillidae | Sweden | NRM 996174 | HQ284684 | KF194140 | KF194005 | KX109744 |
|  | *Pinicola subhimachala* | *Pinicola subhimachala* | Fringillidae | Nepal | FMNH 277061 | KF194180 | KF194136 | KF194036 | - |
| MAR7334 | *Carpodacus nipalensis* | *Procarduelis nipalensis* | Fringillidae | Nepal | NME uncat | KF194121 | KF194153 | KF194020 | KX109719 |
| MAR5549 | *Pyrrhoplectes epauletta* | *Pyrrhoplectes epauletta* | Fringillidae | captivity | MTD C63109 | HQ284689 | - | KX109677 | KX109753 |
| Paur6 | *Pyrrhula aurantiaca* | *Pyrrhula aurantiaca* | Fringillidae | India | BMNH 1949.25.3778 | HQ284584 | - | - | **-** |
| MAR6453 | *Pyrrhula erythaca erythaca* | *Pyrrhula erythaca erythaca* | Fringillidae | China |  | HQ284664 | KX109698 | KX109678 | KX109754 |
| T8789 | *Pyrrhula erythaca owstoni* | *Pyrrhula erythaca owstoni* | Fringillidae | Taiwan |  | HQ284656 | MT210132 | **MT277431** | **MT336203** |
| MAR90020 | *Pyrrhula erythrocephala* | *Pyrrhula erythrocephala* | Fringillidae | Nepal | ZFMK ORN2000.113 | HQ284641 | KX109699 | - | - |
| CMC 36632 | *Pyrrhula leucogenis leucogenis* | *Pyrrhula leucogenis leucogenis* | Fringillidae | Philippines |  | HQ284678 | - | - | - |
| CMC 39085 | *Pyrrhula leucogenis steerei* | *Pyrrhula leucogenis steerei* | Fringillidae | Philippines |  | HQ284679 | - | - | - |
| TB2 | *Pyrrhula murina* | *Pyrrhula murina* | Fringillidae | Portugal |  | HQ284631 | KX109700 | KX109679 | KX109755 |
| Pnip9 | *Pyrrhula nipalensis* | *Pyrrhula nipalensis* | Fringillidae | Taiwan | NMNS 8703 | HQ284674 | KX109701 | KX109680 | KX109756 |
| MAR4749 | *Pyrrhula pyrrhula cineracea* | *Pyrrhula pyrrhula cineracea* | Fringillidae | Russia |  | HQ284627 | MT210135 | **MT277432** | **MT336204** |
| MAR1228 | *Pyrrhula pyrrhula europoea* | *Pyrrhula pyrrhula europoea* | Fringillidae | France |  | HQ284598 | KX109702 | KX109681 | KX109757 |
| MTD-C62580 | *Pyrrhula pyrrhula griseiventris* | *Pyrrhula pyrrhula griseiventris* | Fringillidae | captivity | MTD-C62580 | HQ284623 | MT210134 | **MT277433** | **MT336205** |
| MTDC63200 | *Rhodopechys sanguineus* | *Rhodopechys sanguineus* | Fringillidae | captivity | MTDC63200 | **MT210119** | JN715477 | JN715295 | JN715387 |
| GenBank | *Rhodospiza obsoleta* | *Rhodospiza obsoleta* | Fringillidae | Iran | NRM 20046707 | AF342889 | JN715476 | JN715294 | JN715386 |
| GenBank | *Rhynchostruthus socotranus* | *Rhynchostruthus socotranus* | Fringillidae | Yemen | NRM 570794 | - | JN715478 | JN715296 | JN715388 |
| GenBank | *Alario alario* | *Serinus alario* | Fringillidae | South Africa |  | L76276 | - | - | - |
| GenBank | *Serinus canaria* | *Serinus canaria* | Fringillidae | Captivity | NRM 20026502 | L76266 | JN715484 | JN715301 | JN715393 |
| GenBank | *Serinus canicollis* | *Serinus canicollis* | Fringillidae | Captivity | NRM 20076189 | L78706 | JN715480 | JN715298 | JN715390 |
| GenBank | *Serinus flavivertex* | *Serinus flavivertex* | Fringillidae | Kenia |  | L76295 | - | - | - |
| GenBank | *Serinus pusillus* | *Serinus pusillus* | Fringillidae | Iran | NRM 20046715 | L77873 | JN715488 | JN715305 | JN715397 |
| GenBank | *Serinus serinus* | *Serinus serinus* | Fringillidae | Sweden | NRM 20046491 | L76263 | JN715490 | JN715307 | JN715399 |
| GenBank | *Serinus syriacus* | *Serinus syriacus* | Fringillidae | Israel | NRM 570786 | AY570547 | JN715493 | JN715310 | JN715402 |
| GenBank | *Spinus atratus* | *Spinus atratus* | Fringillidae | Argentina | NRM 546071 | L76385 | JN715411 | JN715229 | JN715319 |
| GenBank | *Spinus atriceps* | *Spinus atriceps* | Fringillidae | Guatemala |  | AF342863 | - | - | - |
| GenBank | *Spinus barbatus* | *Spinus barbatus* | Fringillidae | Argentina | NRM 546142 | L77868 | JN715412 | JN715230 | JN715320 |
| GenBank | *Spinus crassirostris* | *Spinus crassirostris* | Fringillidae | Argentina |  | L77869 | - | - | - |
| GenBank | *Spinus cucullatus* | *Spinus cucullatus* | Fringillidae | captivity | NRM 20026508 | L76299 | JN715416 | JN715234 | JN715324 |
| GenBank | *Spinus dominicensis* | *Spinus dominicensis* | Fringillidae | DR |  | AF342864 | - | - | - |
| GenBank | *Spinus lawrencei* | *Spinus lawrencei* | Fringillidae | USA |  | L76392 | - | - | - |
| GenBank | *Spinus magellanicus* | *Spinus magellanicus* | Fringillidae | Paraguay | NRM 986696 | U79016 | JN715420 | JN715238 | JN715328 |
| GenBank | *Spinus notatus* | *Spinus notatus* | Fringillidae | Mexico |  | U79019 | - | - | - |
| GenBank | *Spinus olivaceus* | *Spinus olivaceus* | Fringillidae | Peru |  | L77871 | - | - | - |
| GenBank | *Spinus pinus* | *Spinus pinus* | Fringillidae | USA | NRM 20016375 | U79020 | JN715422 | JN715240 | JN715330 |
| GenBank | *Spinus psaltria* | *Spinus psaltria* | Fringillidae | USA | NRM 20016376 | L76390 | JN715423 | JN715241 | JN715331 |
| GenBank | *Spinus spinescens* | *Spinus spinescens* | Fringillidae | Venezuela |  | U79017 | - | - | - |
| GenBank | *Spinus spinus* | *Spinus spinus* | Fringillidae | Sweden | NRM 986184 | L76391 | JN715426 | JN715244 | JN715334 |
| GenBank | *Serinus thibetanus* | *Spinus thibetanus* | Fringillidae | Myanmar | BMNH 1948.34.64 | L76279 | JN715494 | JN715311 | JN715403 |
| GenBank | *Spinus tristis* | *Spinus tristis* | Fringillidae | USA | NRM 20016378 | U79022 | JN715427 | JN715245 | JN715335 |
| GenBank | *Spinus xanthogastrus* | *Spinus xanthogastrus* | Fringillidae | Costa Rica |  | L76389 | - | - | - |
| GenBank | *Spinus yarellii* | *Spinus yarellii* | Fringillidae | Brasil |  | U83200 | - | - | - |
| GenBank | *Sturnella superciliaris* | *Sturnella superciliaris* | Icteridae | Paraguay | NRM 996695 | EU325781 | JN715406^1)^ | KC007739^5)^ | EU325839 |
| GenBank | *Anthus trivialis* | *Anthus trivialis* | Motacillidae | Sweden | NRM 976393 | AY228048 | GU816850 | AY228285 | GU816919 |
| GenBank | *Motacilla citreola* | *Motacilla citreola* | Motacillidae |  | UWBM66456 | AF526442 | KJ455509 | KJ454838 | KJ455837 |
| GenBank | *Motacilla alba* | *Motacilla alba* | Motacillidae | Sweden | NRM 976193 | AY228069 | GU816849 | AY228307 | GU816918 |
| GenBank | *Arachnothera longirostra* | *Arachnothera longirostra* | Nectariniidae | Singapore | UWBM81981 | KJ456200 | KJ455330 | KJ454756 | EU680706 |
| GenBank | *Arachnothera magna* | *Arachnothera magna* | Nectariniidae | Myanmar | USNM631801 | KJ456201 | KJ455331 | KJ454757 | KJ455723 |
| GenBank | *Cinnyris asiaticus* | *Cinnyris asiaticus* | Nectariniidae |  |  | KJ456232 | KJ455380 | KJ454777 | KJ455751 |
| GenBank | *Dendroica virens* | *Dendroica virens* | Parulidae | El Salvador | NRM 20066318 | EU325770 | - | GU932316 | EU325828 |
| GenBank | *Parula pitiayumi* | *Parula pitiayumi* | Parulidae | Paraguay | NRM 947170 | AY228071 | JN715407 | AY228309 | JN715315 |
| GenBank | *Ammodramus humeralis* | *Ammodramus humeralis* | Passerellidae | Paraguay | NRM 976701 | EU325784 | GU816853^1)^ | JX515370 | EU325842 |
| GenBank | *Junco hyemalis* | *Junco hyemalis* | Passerellidae | USA | NRM 20016363 | EU325787 | - | KC007686^5)^ | EU325845 |
| GenBank | *Passerculus sandwichensis* | *Passerculus sandwichensis* | Passerellidae | Canada | NRM 20036550 | EU325786 | - | KC007687^5)^ | EU325844 |
| GenBank | *Zonotrichia albicollis* | *Zonotrichia albicollis* | Passerellidae | USA | FMNH380394 | KJ456513 | KJ455699 | KJ454941 | KJ455958 |
| GenBank | *Zonotrichia leucophrys* | *Zonotrichia leucophrys* | Passerellidae | USA | NRM 20036310 | EU325785 | - | KC007685^5)^ | EU325843 |
| MAR2212 | *Montifringilla adamsi* | *Montifringilla adamsi* | Passeridae | Nepal |  | **MN337349** | **MN337357** | **MN337368** | **MN337374** |
| MAR1775 | *Montifringilla blanfordi* | *Pyrgilauda blanfordi* | Passeridae | China |  | **MN337350** | MN337358 | MN337366 | - |
| MAR2093 | *Montifringilla davidiana* | *Pyrgilauda davidiana* | Passeridae | China |  | **MN337351** | **MN337359** | MN337367 | **MN337375** |
| MAR2004 | *Montifringilla henrici* | *Montifringilla henrici* | Passeridae | China |  | DQ244059 | **MN337360** | **MN337369** | **MN337376** |
| MTD_2009_160 | *Montifringilla nivalis nivalis* | *Montifringilla nivalis nivalis* | Passeridae | Italy | MTD C64406 | KX109628 | KX109703 | KX109668 | KX109742 |
| MAR3111 | *Montifringilla nivalis groumgrzimaili* | *Montifringilla nivalis groumgrzimaili* | Passeridae | Mongolia |  | **MN337353** | **MN337362** | **MN337371** | **MN337378** |
| MAR2206 | *Montifringilla ruficollis* | *Pyrgilauda ruficollis* | Passeridae | China |  | **MN337354** | MN337363 | AY228306 | GU816915 |
| MAR426 | *Montifringilla tazcanowskii* | *Onychostruthus tazcanowskii* | Passeridae | China |  | **MN337355** | **MN337364** | **MN337372** | **MN337380** |
| MAR8787 | *Passer ammodendri* | *Passer ammodendri* | Passeridae | China |  | **MT210107** | MT210145 | **MT277434** | **MT336206** |
| UWBM95153 | *Passer diffusus* | *Passer diffusus* | Passeridae | South Africa |  | **MT210109** | MT210144 | **MT277435** | **MT336207** |
| MTD_2008-80 | *Passer domesticus* | *Passer domesticus* | Passeridae | Germany | MTD C64358 | KX109629 | KX109704 | KX109669 | KX109743 |
| 2012-202 | *Passer eminibey* | *Passer eminibey* | Passeridae | captivity |  | **MT210111** | **-** | **MT277436** | **MT336208** |
| GenBank | *Passer flaveolus* | *Passer flaveolus* | Passeridae | Vietnam |  | AF230907 | - | - | - |
| ZMUC117473 | *Passer griseus gongoensis’* | *Passer gongoensis* | Passeridae | Kenya |  | **MT210112** | MT210140 | **MT277437** | **MT336209** |
| GenBank | *Passer griseus griseus* | *Passer griseus griseus* | Passeridae | Senegal |  | AF230908 | - | - | - |
| IPMB9505 | *Passer hispaniolensis* | *Passer hispaniolensis* | Passeridae | Spain |  | **MT210113** | **MN488960** | **MT277438** | **MT336210** |
| MAR4076 | *Passer iagoensis* | *Passer iagoensis* | Passeridae | Cape Verde |  | **MT210104** | MT210136 | **MT277439** | **MT336211** |
| ITA1 | *Passer italiae* | *Passer italiae* | Passeridae | Italy |  | **MT210114** | **KX370756** | **MT277440** | **MT336212** |
| GenBank | *Passer luteus* | *Passer luteus* | Passeridae | Nigeria | NRM 20106041 | AY495394 | GU816846 | GU816938 | GU816913 |
| MAR7031 | *Passer melanurus* | *Passer melanurus* | Passeridae | China |  | **MT210106** | MT210142 | **MT277441** | **MT336213** |
| UWBM95160 | *Passer motitensis* | *Passer moitensis* | Passeridae | South Africa | UWBM95160 | **MT210110** | MT210147 | **MT277442** | **MT336214** |
| GenBank | *Passer montanus* | *Passer montanus* | Passeridae | Sweden | NRM 976359 | AY228073 | GU816845 | AY228311 | DQ785937 |
| MAR6957 | *Passer rutilans* | *Passer rutilans* | Passeridae | China |  | **MT210105** | MT210143 | **MT277443** | **MT336215** |
| GenBank | *Petronia dentata* | *Gymnoris dentata* | Passeridae |  |  | **-** | AF407041 | - | - |
| UWBM66486 | *Petronia petronia brevirostris* | *Petronia petronia brevirostris* | Passeridae | Mongolia | UWBM66486 | **MT210108** | MT210141 | **MN337373** | **MN337381** |
| GenBank | *Petronia petronia petronia* | *Petronia petronia petronia* | Passeridae |  |  | AY228074 | - | - | - |
| GenBank | *Petronia superciliaris* | *Gymnoris superciliaris* | Passeridae |  |  | KJ456382 | KJ455547 | KJ454861 | KJ455859 |
| GenBank | *Petronia xanthocollis* | *Gymnoris xanthocollis* | Passeridae |  |  | KF289836 | - | - | - |
| GenBank | *Peucedramus taeniatus* | *Peucedramus taeniatus* | Peucedramidae |  | NRM20066342 | KJ187057 | KJ187052 | KJ187059 | KJ187072 |
| MAR4859 | *Calcarius lapponicus* | *Calcarius lapponicus* | Plectrophenacidae | Russia |  | KP877751 | KX109706 | KP877859 | KX109717 |
| GenBank | *Calcarius ornatus* | *Calcarius ornatus* | Plectrophenacidae |  | BMNH# (jk94-187) | - | EF529819 | - | - |
| GenBank | *Calcarius pictus* | *Calcarius pictus* | Plectrophenacidae |  |  | DQ489368 | - | - | - |
| MTD TC591 | *Plectrophenax nivalis* | *Plectrophenax nivalis* | Plectrophenacidae | captivity | MTD C63199 | KP877807 | KX109705 | KP877883 | KX109745 |
| GenBank | *Rhynchophanes mccownii* | *Rhynchophanes mccownii* | Plectrophenacidae |  | BMNH# (jk94-74) | - | EF529820 | - | - |
| GenBank | *Ploceus philippinus* | *Ploceus philippinus* | Ploceidae | Singapore | UWBM83556 | KJ456411 | KJ455583 | KJ454870 | KJ455878 |
| GenBank | *Promerops cafer* | *Promerops cafer* | Promeropidae |  | MVZ RB681 | - | DQ125990 | EU680615 | EU680755 |
| GenBank | *Promerops gurneyi* | *Promerops gurneyi* | Promeropidae | South Africa | UWBM70395 | - | GU816832 | GU816929 | GU816901 |
| MAR1607 | *Prunella atrogularis* | *Prunella atrogularis* | Prunellidae | Kazakhstan | MTD C63037 | KX109630 | KX109707 | - | - |
| GenBank | *Prunella collaris erythropygia* | *Prunella collaris erythropygia* | Prunellidae | Russia | RYA2451 | **-** | KC759306 | **-** | **-** |
| UWBM64749 | *Prunella collaris montana* | *Prunella collaris montana* | Prunellidae | Russia | UWBM64749 | KJ456427 | KJ455601 | - | KJ455890 |
| MAR3649 | *Prunella collaris tibetana* | *Prunella collaris tibetana* | Prunellidae | China |  | KX109631 | KX109708 | KX109670 | KX109746 |
| GenBank | *Prunella fagani* | *Prunella fagani* | Prunellidae | PR Yemen | BMNH 1965.M.10598 | - | KC759264 | - | - |
| MAR7548 | *Prunella fulvescens* | *Prunella fulvescens* | Prunellidae | China |  | KX109632 | KX109709 | KX109671 | KX109747 |
| MAR2447 | *Prunella himalayana* | *Prunella himalayana* | Prunellidae | Russia |  | KX109633 | KX109710 | KX109672 | KX109748 |
| MAR6443 | *Prunella immaculata* | *Prunella immaculata* | Prunellidae | China |  | KX109634 | KX109711 | KX109673 | KX109749 |
| GenBank | *Prunella koslowi* | *Prunella koslowi* | Prunellidae | Mongolia | KU20376 | - | KC759271 | - | - |
| MAR1978 | *Prunella modularis* | *Prunella modularis* | Prunellidae | Germany |  | KX109635 | KX109712 | KX109674 | KX109750 |
| GenBank | *Prunella montanella* | *Prunella montanella* | Prunellidae |  |  | NC_027284 | NC_027284 | - | - |
| GenBank | *Prunella ocularis* | *Prunella ocularis* | Prunellidae | Armenia | IVF 1092 | - | KC759291 | - | - |
| MAR3636 | *Prunella rubeculoides* | *Prunella rubeculoides* | Prunellidae | China |  | KX109636 | KX109713 | KX109675 | KX109751 |
| GenBank | *Prunella rubida* | *Prunella rubida* | Prunellidae | Russia | UWBM 45240 | - | KC759305 | - | - |
| MAR6616 | *Prunella strophiata* | *Prunella strophiata* | Prunellidae | Nepal | NME 08/006 | KX109637 | KX109714 | KX109676 | KX109752 |
| GenBank | *Camarhynchus pallidus* | *Camarhynchus pallidus* | Thraupidae | Ecuador |  | KJ945365 | - | - | KJ945566 |
| GenBank | *Camarhynchus parvulus* | *Camarhynchus parvulus* | Thraupidae | Ecuador |  | KJ945385 | - | - | KJ945586 |
| GenBank | *Camarhynchus pauper* | *Camarhynchus pauper* | Thraupidae | Ecuador |  | KJ945341 | - | - | KJ945543 |
| GenBank | *Camarhynchus psittacula* | *Camarhynchus psittacula* | Thraupidae | Ecuador |  | KJ945364 | - | - | KJ945565 |
| GenBank | *Certhidea fusca* | *Certhidea fusca* | Thraupidae | Ecuador |  | KJ945353 | - | - | KJ945555 |
| GenBank | *Certhidea olivacea* | *Certhidea olivacea* | Thraupidae | Ecuador |  | KJ945386 | - | - | KJ945587 |
| GenBank | *Coereba flaveola* | *Coereba flaveola* | Thraupidae | Bahamas | BMNH#JK95001 | AF290151 | AF290114 | GU932350 | EU325838 |
| GenBank | *Geospiza conirostris* | *Geospiza conirostris* | Thraupidae | Ecuador |  | KJ945343 | - | - | KJ945545 |
| GenBank | *Geospiza difficilis* | *Geospiza difficilis* | Thraupidae | Ecuador |  | KJ945383 | - | - | KJ945585 |
| GenBank | *Geospiza fortis* | *Geospiza fortis* | Thraupidae | Ecuador |  | KJ945358 | - | - | KJ945560 |
| GenBank | *Geospiza fuliginosa* | *Geospiza fuliginosa* | Thraupidae | Ecuador |  | KJ945377 | - | - | KJ945579 |
| GenBank | *Geospiza magnirostris* | *Geospiza magnirostris* | Thraupidae | Ecuador |  | KJ945349 | - | - | KJ94555 |
| GenBank | *Geospiza scandens* | *Geospiza scandens* | Thraupidae | Ecuador |  | KJ945370 | - | - | KJ945571 |
| GenBank | *Pinaroloxias inornata* | *Pinaroloxias inornata* | Thraupidae | Ecuador |  | KJ945356 | - | - | KJ945558 |
| GenBank | *Platyspiza crassirostris* | *Platyspiza crassirostris* | Thraupidae | Ecuador |  | KJ945378 | - | - | KJ945580 |
| GenBank | *Thraupis palmarum* | *Thraupis palmarum* | Thraupidae | Tobago | NRM 20076366 | EU325779 | EU648105 | JN810421 | EU325837 |
| MAR3616 | *Urocynchramus pylzowi* | *Urocynchramus pylzowi* | Urocynchramidae | China | MTD C64770 | KX109639 | KX109715 | KX109682 | KX109758 |
| Non-passerine outgroups | | | | | | | | | |
|  | *Falco subbuteo* | *Falco subbuteo* | Falconidae |  |  | EU233117 | GU816825 | JN614732 | JN614588 |
|  | *Nestor notabilis* | *Nestor notabilis* | Strigopidae |  |  | AF346389 | EU327641 | KY763610 | - |
|  | *Cacatua sulphurea* | *Cacatua sulphurea* | Cacatuidae |  |  | AB177976 | JF414345 | EU739932 | DQ881722 |
|  |  |  |  |  |  |  |  |  |  |

**Table S3** Primer combinations used for PCR and sequencing; ^1)^ primer used only for sequencing; t= annealing temperature.

| **cytochrome *b*** |  | **Forward** |  | **reverse** |  |  |
| --- | --- | --- | --- | --- | --- | --- |
|  | Primer | Sequence (5'-->3') | primer | Sequence (5'-->3') | **PCR settings** |  |
|  | O-L14851 | CCTACCTAGGATCATTCGCCCT | O-H16065 | AGTCTTCAATCTTTGGCTTACAAGAC | 94°C for 5 min  35 cycles  94°C for 45 s  53 °C for 45 s  72°C 1:30 min  72°C for 7 min | standard |
|  | | | | | | |
| **ND2** |  | **Forward** |  | **reverse** |  |  |
| PCR, external | L5216 | GGCCCATACCCCGRAAATG | H6361 | ACTCTTRTTTAAGGCTTTGAAGGC | same settings as cyt*b* | standard |
| Internal | L5758 | GGNGGNTGAATRGGNYTNAAYCARAC | H5766 | RGAKGAGAARGCYAGGATYTTKCG |  |  |
| PCR, external | Passer_ND2F | GGCCCATACCCCGRAAATG | Passer_ND2R | ACTCTTRTTTAAGGCTTTGAAGGC | same settings as cyt*b* | sparrows |
| internal^1)^ | PasserND2_seqintF | ACCATCACTAAATCCCACACTC | PasserND2_seqintR | TAAGGTGAGGAAGACTGTTGAG |  |  |
|  | | | | | | |
| **Myo** |  | **forward** |  | **reverse** |  |  |
| PCR, external | myo2 | CGG AAG AGC TCC AGG GCC TT | myo3 | GCCACCAAGCACAAGATCCC | touchdown PCR  95°C for 4 min  total 35 cycles  95°C for 1 min  [60°C for 1min – 5 cycles  58°C for 1 min – 5 cyc]es  56°C for 1 min – 25 cycles]  72°C 1:30 min | standard |
| internal^1)^ |  |  | myo3F | GCAAGGACCTTGATAATGACTT |  |  |
|  | | | | | | |
| **ODC** |  | **forward** |  | **reverse** |  |  |
| PCR, external | OD6 | GACTCCAAAGCAGTTTGTCGTCTCAGTGT | OD8R | TCTTCAGAGCCAGGGAAGCCACCACCAAT | Same as myoglobin | standard |
| PCR, external | OD6mod | GGGGCTACACTTAAGACCAG | OD8mod | TCTTCAGAGCCAGGGAAGCCACCACCAAT | Same as myoglobin | buntings  rosefinches |
| internal^1)^ | OD6int | GTTCTACTTTCTCTGGAACTACTGCTC | OD8int | CGCYTWAACAYTCYGCCATCTTACC |  |  |

**Table S4** Calibration points used for molecular dating and tmrca prior settings in BEAST (modified from Päckert et al., 2016); five lognormal priors (including zero offset = minimum node age ( = fossil age), log mean and standard deviation (sd)), and three normal priors; tmrca priors for fossil calibration points were set to a lognormal distribution roughly covering the interval of the stratigraphic layer with the fossil age as zero offset (see Benton et al*.*, 2009).

| **node dated** | **calibration source** | **type** | **date (Ma)** | **initial value** | **offset** | **mean** | **log mean** | **sd** | **reference** |
| --- | --- | --- | --- | --- | --- | --- | --- | --- | --- |
| *Nestor* + *Cacatua*  ( = Strigopidae vs. Cacatuidae) | secondary calibration derived from several fossil calibrations (according to Jetz et al*.*, 2014) | lognormal | 54 | 54 | 54 | – | 2.7 | 0.6 | Rheindt et al*.*, 2013 |
| all passerines | biogeographic: separation of New Zealand from Australia^1)^; fossil: earliest crown group passerines from Australia^2)^ | lognormal | 30–85 | 54 | 30 | – | 1.0 | 0.5 | 1) Kennedy et al*.*, 2012;  2) Mayr 2013 |
| *Cyanocompsa cyanoides* | fossil: *Passerina*, Hemphillian | lognormal | 4.5–10 | 4.5 | 4.5 | – | 0.75 | 0.8 | Kennedy et al*.*, 2012;  Steadman & McKitrick 1982 |
| Drepaninidae | biogeographic: formation of Kauai/Nilhau complex | normal | 4.7–5.7 | 4.7 | – | 4.7 | – | 0.25 | Lerner et al*.*, 2011 |
| *C. formosanus/C. vinaceus* | biogeographic: emergence of Taiwan and formation of central mountain ridge | lognormal | 5 | 1 | 0 | – | 0.01 | 0.8161 | Päckert et al*.*, 2012 |
| *P. e. erythaca/P. e. owstoni* | biogeographic: emergence of Taiwan and formation of central mountain ridge | lognormal | 5 | 1 | 0 | – | 0.01 | 0.8161 | Päckert et al*.*, 2012 |
| *Certhidea fusca* vs. crown clade | secondary calibration, origin of Darwin’s finches radiation | normal | 0.9 | 0.9 | 0 | 0.9 | – | 0.123 | Lamichhaney et al., 2015 |
| *P. murina*/*P. pyrrhula* | biogeographic: age of São Miguel | normal | 0–0.88 | 0.9 | 0 | 0.4 | – | 0.47 | Töpfer et al*.*, 2010 |

**Table S5a** Dispersal multiplier matrix as applied to BioGeoBEARS analysis in ARR1; eight areas: A= New World (Nearctic + Neotropics), B= Western Palearctic, C= Afrotropics, D= Central Asia + Middle East, E= QTP, F= Sinohimalayas, G= Eastern Palearctic, H= Oriental Region.

A B C D E F G H

1.0 0.5 0.01 0.5 0.5 0.5 1.0 0.5

0.5 1.0 1.0 1.0 0.5 0.5 1.0 0.01

0.01 1.0 1.0 0.5 0.01 0.01 0.01 0.5

0.5 1.0 0.5 1.0 1.0 1.0 1.0 1.0

0.5 0.5 0.01 1.0 1.0 1.0 1.0 0.5

0.5 0.5 0.01 1.0 1.0 1.0 1.0 1.0

1.0 1.0 0.01 1.0 1.0 1.0 1.0 1.0

0.5 0.01 0.5 1.0 0.5 1.0 1.0 1.0

**Table S5b** Dispersal multiplier matrix as applied to BioGeoBEARS analysis in ARR2; nine areas: same area coding as in ARR1 only Sinohimalayas treated as two separate areas F_1_ (Himalayas) F_2_ (Hengduanshan).

A B C D E F1 F2 G H

1.0 0.5 0.01 0.5 0.5 0.01 0.5 1.0 0.5

0.5 1.0 1.0 1.0 0.5 0.5 0.01 1.0 0.5

0.01 1.0 1.0 0.5 0.01 0.01 0.01 0.5 0.01

0.5 1.0 0.5 1.0 1.0 1.0 0.5 1.0 1.0

0.5 0.5 0.01 1.0 1.0 1.0 1.0 1.0 0.5

0.01 0.5 0.01 1.0 1.0 1.0 1.0 0.5 1.0

0.5 0.01 0.01 0.5 1.0 1.0 1.0 1.0 1.0

1.0 1.0 0.5 1.0 1.0 0.5 1.0 1.0 1.0

0.5 0.5 0.01 1.0 0.5 1.0 1.0 1.0 1.0

**Additional references**

Hou, Y., Bjora, C.S., Ikeda, H., Brochmann, C. & Popp, M. (2016). From the north into the Himalayan–Hengduan Mountains: fossil-calibrated phylogenetic and biogeographical inference in the arctic-alpine genus *Diapensia* (Diapensiaceae). Journal of Biogeography, 43, 1502–1513.

Kennedy, J.D., Weir, J.T., Hooper, D.M., Tietze, D.T., Martens, J. & Price, T. (2012). Ecological limits on diversification of the Himalayan core Corvoidea. Evolution, 66, 2599–2613.

Lamichhaney, S., Berglund, J., Almén, M.S., Maqbool, K., Grabherr, M., Martinez-Barrio, A. (…) Andersson, L. (2017): Evolution of Darwin’s finches and their beaks revealed by genome sequencing. Nature, 518, 371–375.

Lerner, H.R.L., Meyer, M., James, H.F., Hofreiter, M. & Fleischer, R.C. (2011). Multilocus resolution of phylogeny and timescale in the extant adaptive radiation of Hawaiian honeycreepers. Current Biology, 21, 1–7.

Mayr, G. (2013). The age of the crown group of passerine birds and its evolutionary significance – molecular calibrations versus the fossil record. Systematics and Biodiversity, 11, 7–13.

Rheindt, F.E., Christidis, L., Kuhn, S., de Kloet, S., Norman, J.A., & Fidler, A. (2013). The timing of diversification within the most divergent parrot clade. Journal of Avian Biology, 45, 140-148.

Steadman, D.W. & McKitrick, M.C. (1982). A Pliocene bunting from Chihuahua, Mexico. Condor, 84, 240–241.

Tietze, D.T., & Borthakur, U. (2012). Historical biogeography of tits (Aves: Paridae, Remizidae). Organisms, Diversity and Evolution, 12, 433-444.

Zhang, M.L., Meng, H.H., Zhang, H.X., Vyacheslav, B.V. & Sanderson, S.C. (2014). Himalayan origin and evolution of *Myricaria* (Tamaricacaeae) in the Neogene*. PLoS One, 9,* e97582.
